# Organic Bulk‐Heterojunction‐Integrated Flexible Perovskite Photodetection Arrays for High‐Speed Broadband Optical Communication

**DOI:** 10.1002/advs.202509546

**Published:** 2025-12-08

**Authors:** Xin Hong, Kanghui Ke, Yu Gao, Jiazhi Meng, Bohua Deng, Haoyu Huang, Zhichun Si, H. Y. Fu, Feiyu Kang, Guodan Wei

**Affiliations:** ^1^ Institute of Materials Science, Tsinghua Shenzhen International Graduate School Tsinghua University Shenzhen 518000 China; ^2^ Tsinghua Shenzhen International Graduate School and Tsinghua‐Berkeley Shenzhen Institute (TBSI) Tsinghua University Shenzhen 518055 China

**Keywords:** flexible photodetector, NIR imaging, organic bulk heterojunction, rapid response, ultralow dark current

## Abstract

The demand for self‐powered, broadband photodetectors is rising in fields like artificial intelligence, health monitoring, and optical communications. However, conventional perovskite photodetectors face limited visible absorption and poor ambient stability. Here, we report a high‐performance integrated photodetector combining a perovskite (Cs_n.15_FA_n.85_PbI_3_) absorber with an organic bulk‐heterojunction (BHJ) structure for broadband photon harvesting up to 1000 nm. The device achieves a peak external quantum efficiency (EQE) of 84% in the visible range (400–700 nm) and 63% in the near‐infrared (NIR, 700–1000 nm). Benefiting from optimized energy band alignment, the photodetector exhibits a self‐powered responsivity of 0.3 A W^−1^ and a fast response time of 29 µs, with a linear dynamic range (LDR) of 122 dB under 900 nm NIR illumination. The BHJ organic layer suppresses the dark current (J_D_) to 6.9 × 10^−11^ A cm^−2^ and noise current (i_n_) to 10‐14 A Hz^−1/2^, yielding a specific detectivity (D*) of 10^12^ Jones. The polymeric BHJ also enhances flexibility, enabling a reliable 25‐pixel array for uniform NIR imaging. In optical communication, the device achieves a low bit error rate with data rates of 90 kbps (λ = 665 nm) and 140 kbps (λ = 904 nm). This work establishes the perovskite/BHJ detector as a promising platform for flexible, high‐speed optoelectronics.

## Graphical abstract

A flexible broadband photodetector is realized by integrating a perovskite with an organic bulk‐heterojunction. It achieves high external quantum efficiency (EQE) (>80% visible, >60% near‐infrared (NIR)), ultralow dark current, and fast response, enabling self‐powered, high‐detectivity operation. The device facilitates robust NIR imaging, health monitoring, and high‐speed optical communication on a flexible platform.

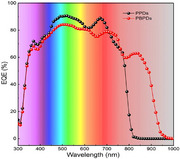

## 1 Introduction

Broadband photodetectors (PDs) are integral to a variety of disciplines, ranging from remote sensing and artificial intelligence to health diagnostics, optical data transmission, and persistent surveillance.^[^ ^1^, ^2^, ^3^, ^4^ ^]^ Predominantly, these PDs have relied upon inorganic semiconductors—Specifically, gallium nitride (GaN) demonstrates optimal UV responsiveness, silicon (Si) excels in visible light detection, and indium gallium arsenide (InGaAs) dominates near‐infrared (NIR) sensing—owing to their broad wavelength detection capabilities spanning from UV to NIR spectra.^[^ ^5^, ^6^, ^7^, ^8^ ^]^ However, the intrinsic inflexibility of these materials has limited their compatibility with emerging flexible electronics.^[^ ^9^ ^]^ This limitation has spurred the pursuit of alternative semiconducting materials such as perovskites and organic compounds, which possess superior optical absorption efficiencies, tunable electronic band structures, and processing versatility.^[^ ^10^, ^11^, ^12^, ^13^ ^]^ These materials' amenable mechanical properties further facilitate their incorporation into flexible substrates, heralding a new breed of photodetectors. These fast‐developing devices not only exhibit enhanced sensitivity and reduced mass but also maintain structural integrity under mechanical deformation, essential characteristics for the next generation of photodetection technology.^[^ ^14^, ^15^, ^16^ ^]^

The integration of perovskite and organic semiconductors is transforming traditional detection modes, paving the way for a new era of versatile, broadband PDs with superior imaging capabilities. Lead‐based perovskite photodetectors have set benchmarks for their rapid response time, high specific detectivity (*D* ^*^), and expansive linear dynamic range (*LDR*) within the visible spectrum.^[^ ^17^ ^]^ However, when it comes to the NIR region, they fall short beyond 800 nm due to their inherently wide bandgap. Modifying the perovskite structure by substituting Pb^2+^ with Sn^2+^ has shown promise, extending absorption to nearly 900 nm.^[^ ^13^, ^18^ ^]^ However, this substitution is fragile with challenges as Sn^2+^ is prone to oxidation under normal environmental conditions, leading to compromised film quality and device instability.^[^ ^19^, ^20^ ^]^ Organic semiconductor materials, on the other hand, exhibit various molecular designs and remarkable flexibility, positioning them as prime candidates for efficient NIR photodetection.^[^ ^21^, ^22^ ^]^ A synergetic approach that combines the wide‐bandgap properties of perovskites for visible light with the narrow‐bandgap characteristics of organic materials for NIR light has been envisioned, harmonizing the spectral capabilities of both inorganic and organic materials. For example, a composite photodetector with MAPbI_3_ and PDPPTDTPT/PC_61_BM active layers has effectively pushed the detection threshold to 950 nm.^[^ ^23^ ^]^ Furthermore, the introduction of PTB7‐Th:F8IC as an organic bulk heterojunction on top of MAPbI_3_ layer has resulted in an extensive external quantum efficiency (*EQE*) covering 1000 nm, peaking at 54 % in the NIR region alongside an impressive detectivity of over 2.3 × 10^11^ *Jones* (cm Hz^1/2^ W^−1^) at 870 nm.^[^ ^24^ ^]^ The latest development synergistically combine perovskite with PM6: BTP‐4F organic layers, exhibiting broad detection ranges for both visible and NIR light, alongside shortened response times and a significantly broadened linear dynamic range of 143 dB.^[^ ^25^ ^]^ Despite these advances, it remains critical to optimize *EQE* values further across the spectrum and suppress the dark current with refined device architectures. The versatility and potential for flexibility in these detectors are key considerations for their application in wearable electronics.^[^ ^26^, ^27^ ^]^ Our integration strategy, utilizing perovskite/NIR organic bulk heterojunctions (BHJ), proposes an expansion of the detection capabilities from the visible to NIR spectrum. This is achieved through a vertically stacked device structure processed with orthogonal solvents.^[^ ^28^ ^]^ Our prior work successfully established double cascading charge transfer pathways within the perovskite/BHJ interfaces, enhancing NIR light capture to 950 nm and significantly amplifying the photocurrent.^[^ ^29^ ^]^ Furthermore, the perovskite/organic BHJ dual‐band photodetectors have demonstrated a swift and switchable photoresponse. These devices achieve peak responsivities reaching 93.5 mA W^−1^ for the visible spectrum at a wavelength of 625 nm when a forward bias of 0.7 V is applied, and an even higher responsivity of 102.2 mA W^−1^ in the NIR spectrum at a wavelength of 900 nm under a reverse bias of −1.5 V.^[^ ^30^ ^]^

In this work, broadband visible‐NIR PDs were constructed using an integrated Cs_0.15_FA_0.85_PbI_3_ perovskite/organic BHJ architecture. The Cs_0.15_FA_0.85_PbI_3_ perovskite was deposited as the bottom layer on an ITO substrate, with the organic BHJ layer formed on top. For the BHJ, we employed a narrow bandgap acceptor (BTP‐4F)^[^ ^31^ ^]^ and a conjugated polymer donor (D18)^[^ ^32^ ^]^ to construct an efficient donor‐acceptor system. In addition, a fullerene derivative (PC_61_BM) was incorporated to form a ternary BHJ, enhancing charge mobility and overall film conductivity. This combination of perovskite and organic semiconductors‐with complementary absorption profiles‐resulted in an extended spectral response up to 1000 nm. The integrated PD achieved a peak *EQE* of 84% in the visible region and 63% in the NIR range. Importantly, the introduced organic BHJ significantly suppressed the dark current density (*J*_D_) by over three order of magnitudes‐from 10^−8^ A cm^−2^ down to 10^−11^ A cm^−2^ and reduced the noise current from 10^−12^ down to 10^−14^ A Hz^−1/2^, compared to pristine perovskite devices. These enhancements enabled a high detectivity of 10^12^ *Jones* and linear dynamic range (*LDR*) of 122 dB under 900 nm illumination. Beyond optoelectronic benefits, the BHJ layer serves a dual function: it alleviates mechanical strain, allowing the device to endure repeated bending, and acts as a protective barrier against moisture, thereby improving environmental stability and operational durability. As a result, the flexible photodetector array, consisting of a 25‐pixel grid in a 5 × 5 arrangement, maintains air stability and reliable operation under various illumination conditions. Its effectiveness was demonstrated through successful NIR imaging and contactless heart‐rate monitoring, highlighting its suitability for integration into wearable electronics. Moreover, in optical communication scenarios, our photodetector maintained a low bit error rate across visible (λ = 665 nm) and NIR (λ = 904 nm) illumination, providing its capacity to reliably transmit both images and textual data at rates of 90 and 140 kbps, respectively. Thus, this flexible photodetector, characterized by an extended NIR detection range and enhanced mechanical resilience, offers a promising platform for next‐generation health monitoring technologies, including pulse oximetry and medical imaging as well as high‐speed optical communications.

## 2 Results and Discussion

**Figure 1:**
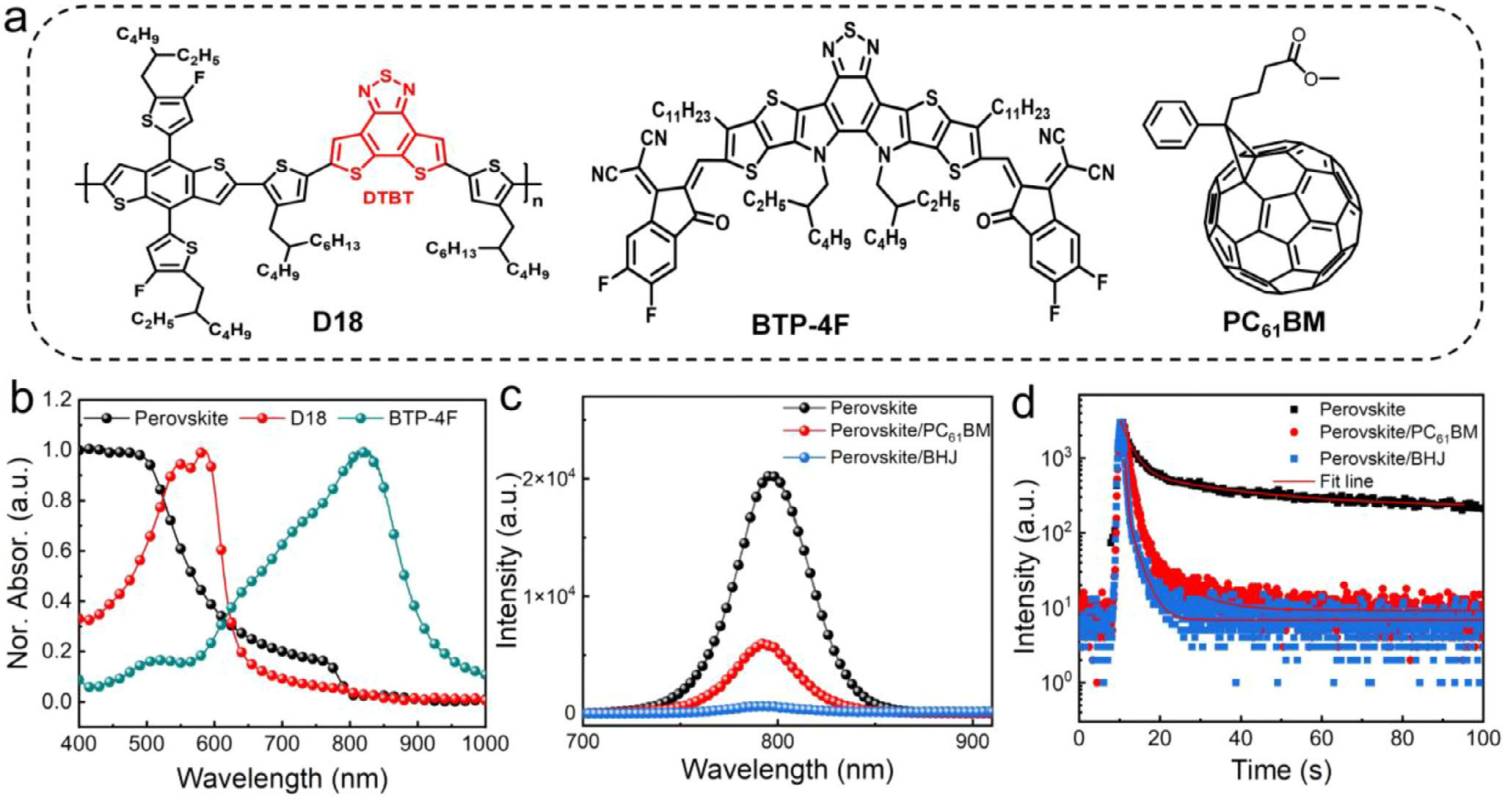
Basic characteristics of the perovskite and BHJ film. a) Structural formulae of D18, BTP‐4F and PC_61_BM. b) UV–visible solid‐state spectrum of various thin films, including perovskite, D18 and BTP‐4F. c) PL and (d) TRPL of pristine perovskite, perovskite films coated with the PC_61_BM, and BHJ layer, respectively at 797 nm.

The structural formulae of D18, BTP‐4F and PC_61_BM in the BHJ are shown in **Figure** **1**a. The absorption spectra of the pristine perovskite, D18, BTP‐4F film are shown in Figure 1b, where a typical absorption edge of Cs_0.15_FA_0.85_PbI_3_ perovskite is observed at ≈800 nm. The donor material, D18, covers the visible range absorption and the narrow bandgap semiconductor, BTP‐4F, has the absorption range in NIR to extend edge to 1000 nm. Steady‐state photoluminescence (PL) and time‐resolved PL (TRPL) spectra are acquired to explore the carrier extraction process at the perovskite/ (PC_61_BM:D18:BTP‐4F) ternary BHJ interface. Figure 1c shows that the perovskite film has a PL emission peak at 797 nm. After being coated with the PC_61_BM layer, an efficient PL quenching is observed and the PL emission intensity is further reduced after adding the BHJ layer due to more efficient charge transfer process. TRPL results also showcase a rapid carrier extraction process (Figure 1d). We conducted fitting of the fluorescence lifetime of the thin film at 800 nm using the following second‐order equation^[^ ^33^ ^]^:

(1) $$y=y_{0}+A_{1}e^{-x/\tau_{1}}+A_{2}e^{-x/\tau_{2}}$$

Generally, the TRPL lifetime can be divided into two parts: (1) the fast decay lifetime (τ_1_) corresponding to the carrier bimolecular recombination at the interface; and ([]2) the slow decay lifetime (τ_2_) corresponding to the radiative recombination of free carriers.^[^ ^33^, ^34^, ^35^ ^]^ The average decay time (τ_avg_) of the pristine perovskite film primarily stems from τ_2_, measured at 105.85 ns due to two‐decay processes (Table S1, Supporting Information). Upon spin‐coating the PC_61_BM layer onto the perovskite, recombination at the interface dominates the rapid decay lifetime (τ_1_), constituting 87.46% of the overall decay process. Subsequent BHJ coating extends τ_1_ to 89.42% while reducing τ_2_ from 1.28 to 0.42 ns, indicating enhanced fast carrier extraction at the perovskite/BHJ interface, which is beneficial for fast photoresponse upon light excitation.

**Figure 2:**
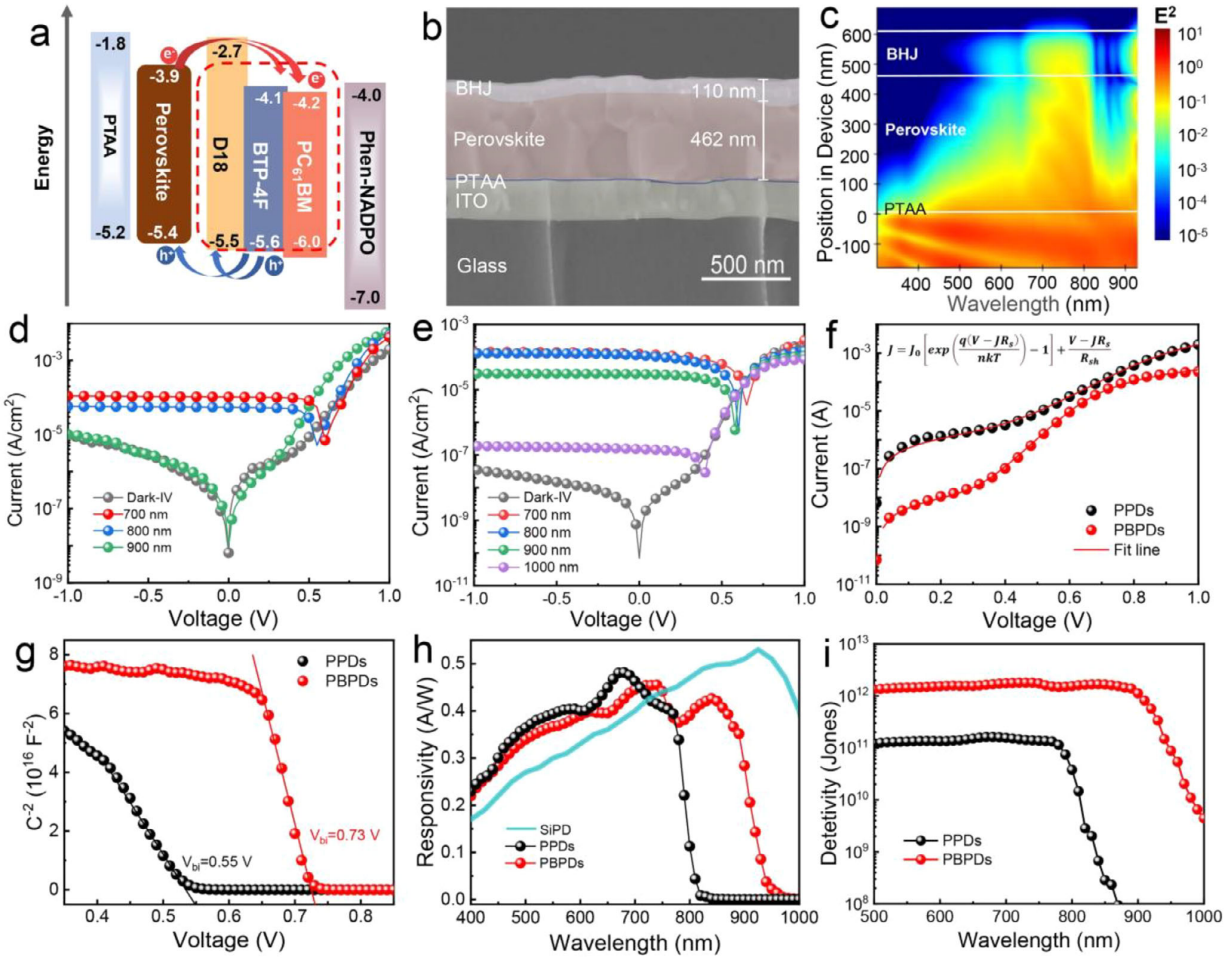
Device performance of PDs. Device structure and energy level diagram a) of PBPDs. b) Cross‐sectional SEM image of the PD except Ag. c) Calculated light field distribution in PBPDs. d,e) *I–V* curves of PPDs and PBPDs under dark and illumination with different wavelengths for a power density of 20 mW cm^−2^. f) Dark Current versus voltage varies from 0 to 1 V of PPDs and PBPDs and fitted results of dark current through revised diode equation. g) Mott‐Schottky plot of PPDs and PBPDs. Responsivity h) and detectivity i) of the PPDs and PBPDs with respect to the wavelength with an applied voltage of 0 V.

The diode device with an inverted structure of ITO/PTAA/perovskite/(PC_61_BM only or BHJ/Phen‐NADPO/Ag) is prepared to characterize the device performance. **Figure** **2**a shows the energy level diagram for each layer. The photoexcitation of perovskite thin films could generate free electron and holes, while the photoexcitation of organic thin films results in the generation of Frenkel excitons,^[^ ^36^ ^]^ causing unbalanced carrier transport and unnecessary non‐radiative recombination.^[^ ^32^, ^37^ ^]^ To overcome these drawbacks, PC_61_BM is added to form a ternary mixture for cascading double charge transport channels, facilitating an efficient electron transport process with BTP‐4F in BHJ.^[^ ^29^, ^38^ ^]^ The cross‐sectional SEM image (Figure 2b) shows that a 462 nm thick perovskite and a 110 nm organic layer are uniformly deposited. The simulated optical field distribution, as depicted in Figure 2c, illustrates the wavelength‐dependent penetration depth across both the perovskite layer and the organic BHJ layer, indicating the potential for achieving absorption in the visible/near‐infrared wavelength range through the combined influence of these two layers. Figure 2d,e are the *I–V* characteristics of PDs based on the perovskite film with PC_61_BM (PPDs) and BHJ(PBPDs) under the dark and illumination at different wavelengths. Both PPDs and PBPDs exhibit high photocurrent in the visible light due to the absorption of perovskite. For PBPDs, the device has a significant response at 900 nm incident light illumination. In addition to the expansion to the NIR range, the PBPDs with BHJ layer also significantly suppresses the dark current density (*J*_D_) with more than three orders of magnitude from 2.6 × 10^−8^ to 6.9 × 10^−11^ A cm^−2^, implying an idealized heterojunction formed to minimize the leakage current (Figure 2e). The significantly suppressed dark current and noise in our PBPDs lead to a markedly improved signal‐to‐noise ratio (SNR), a key metric for photodetection fidelity. Under 900 nm illumination (20 mW cm^−2^), PBPDs exhibit a photocurrent density of ≈10^−^ ^6^ A cm^−2^ against a noise floor of ≈10^−^ ^14^ A Hz^−^^1/2^, yielding an SNR far superior to that of PPDs. This aligns with the established strategy of enhancing detector performance by minimizing the noise level via dark current suppression.^[^ ^39^, ^40^ ^]^ Our perovskite/BHJ integration effectively implements this approach, achieving an ultralow *J*_D_ of 6.9 × 10^−^ ^11^ A cm^−2^ and the resultant high SNR, crucial for the demonstrated high‐fidelity NIR imaging and optical communication. Also, we performed fitting of PPDs and PBPDs using the revised diode equation (Figure 2f), where *J*_0_ represents for dark saturation current, *n* is ideality factor, *R_S_* is series resistance and *R*_*s*h_ is shunt resistance, was applied to investigate different magnitude of dark current of the two devices. Surprisingly, the PBPDs fit well with the revised diode equation with an ideality factor of *n* = 1.62, a value closer to the ideal case of radiative recombination (*n* = 1) than to trap‐assisted non‐radiative recombination (*n* = 2) observed in PPDs (*n* = 2.76), indicating that the nonradiative recombination has been significantly suppressed. Though PBPDs shows larger *R_S_*, potentially stemming from the inferior conductivity of the BHJ layer in comparison to pristine perovskite layer, PBPDs exhibit the *R*_*s*h_ two orders of magnitude higher than the PPDs (Table S2, Supporting Information).

To further quantitatively investigate the impact of integrating the BHJ layer on the charge transfer process within the device, Mott‐Schottky curves were employed to characterize the built‐in electric field of PPDs and PBPDs using Equation (2): ^[^ ^41^ ^]^

(2) $$\frac{1}{C^{2}}=\frac{2}{\varepsilon \varepsilon_{0}eA^{2}N}\left(V_{\mathrm{bi}}-V\right)$$

As depicted in Figure 2g, PBPDs exhibited a higher built‐in potential (*V*_bi_) of 0.73 V compared to the control PSCs with a potential of 0.55 V. Furthermore, the steeper slope of the Mott‐Schottky plot for PBPDs indicates a lower charge carrier density within the depletion region compared to PPDs. This reduction in carrier density is consistent with the significantly suppressed dark current and the higher *V*_bi_, collectively contributing to the enhanced diode characteristics and detection performance.

The external quantum efficiency (*EQE*) of PPDs and PBPDs are shown in Figure S1 (Supporting Information). Due to the complementary absorption of these two layers, the integrated photodetector could dramatically expand the response range until 1000 nm and a peak *EQE* as high as 84% in the visible range, as well as a peak *EQE* of 63% in the NIR range. The photoresponsivity (*R*_λ_) can be calculated by the following equation^[^ ^42^, ^43^ ^]^:

(3) $$R_{\lambda }=\mathrm{EQE}\times \frac{q}{\mathrm{hv}}$$

where *q* is the elementary charge; h*v* is the photon energy; and *R*_λ_ indicates how efficiently the detector responds to an optical signal. Figure 2h shows the expanded NIR response of 0.4 A W^−1^ (700–900 nm) for the perovskite/BHJ device with high responsivity in the visible range (400–700 nm), which is close to commercial silicon photodetectors (SiPDs).^[^ ^44^ ^]^ Therefore, PBPDs could simultaneously realize the advantages of both perovskite and organic layers for high responsivity and broad‐spectrum response, as well as extremely low dark current, indicating perfect complimentary material selection with favorable energy level alignment. The detectivity (D*) of PDs can be calculated based on the noise current (Figure S2, Supporting Information) in the following equation^[^ ^21^, ^45^ ^]^:

(4) $$D^{*}=\frac{\sqrt{A\Delta f}}{\mathrm{NEP}}\frac{\sqrt{A\Delta f}}{i_{n}/R}$$

where *NEP* is the noise equivalent power and *i_n_* is the noise current. According to Equation (4) with the noise density at 1 × 10^3^ Hz, the D* of PPDs is calculated as 10^11^ *Jones*. The noise spectral density of the PBPDs under dark conditions at 0 V bias is shown in Figure S2 (Supporting Information). The flat frequency response at higher frequencies is characteristic of shot‐noise‐limited behavior. The significantly lower noise in PBPDs stems directly from its ultralow dark current (Figure 2e), which is suppressed by the BHJ‐integrated structure. This reduction in dark current minimizes the shot noise floor, thereby enabling a high detectivity D* of 10^12^ Jones. The improvement of D* is greatly attributed to the reduction in the noise current by the organic BHJ layer as well as comparable photocurrent generation upon visible and NIR light excitation. The integrated perovskite/BHJ device shows obvious advantages when compared to PPDs and organic PDs (OPDs). A summary of device performances from recently reported PDs based on perovskite and organic semiconductor are listed in **Table** **1**. The as‐obtained PBPDs have the lowest dark current as well as the highest responsivity and detectivity both at the visible and NIR range, clearly indicating the successful integration of perovskite and organic layers.

**Table 1:** A summary of device performances of some recently reported PDs based on perovskite and organic semiconductor.

Device structure	*R* [mA/W] [600/850 nm]	*J*_D_ [A cm^−2^]	Max *D* ^*^ [*Jones*]	Refs.
ITO/SnO_2_/Pero/PTAA/MoO_3_/Ag	450/–	10^−9^	10^11^	[11]
ITO/PTAA/Pero/PCBM/Ag	500/–	10^−9^	10^12^	[12]
ITO/PTAA/Pero/C60/BCP/Ag	300/–	10^−10^	10^13^	[4]
ITO/TiO_2_/Pero/Spiro/ MoO_3_/Ag	300/–	10^−5^	10^12^	[47]
ITO/CdS/MAPbI_3_/Au	400/–	10^−8^	10^11^	[48]
ITO/PEDOT:PSS/BHJ/Phen‐NADPO/Ag	400/390	10^−10^	10^13^	[40]
ITO/HTL/BHJ/ETL/Ag	380/300	10^11^	10^12^	[49]
ITO/ZnO_x_/BHJ/MoO_x_/Ag	300/450	10^−10^	10^13^	[50]
ITO/ZnO_x_/BHJ/MoO_x_/Ag	400/200	10^10^	10^11^	[51]
ITO/HTL/D:A/ETL/Ag	320/450	10^−9^	10^11^	[52]
ITO/PTAA/Pero/BHJ/BCP/Ag	380/270	10^−9^	10^11^	[23]
ITO/PTAA/Pero/BHJ/C60/BCP/Ag	350/360	10^−9^	10^11^	[24]
ITO/PTAA/Pero/BHJ/Phen‐NADPO/Ag	460/400	10^−11^	10^12^	This work

**Figure 3:**
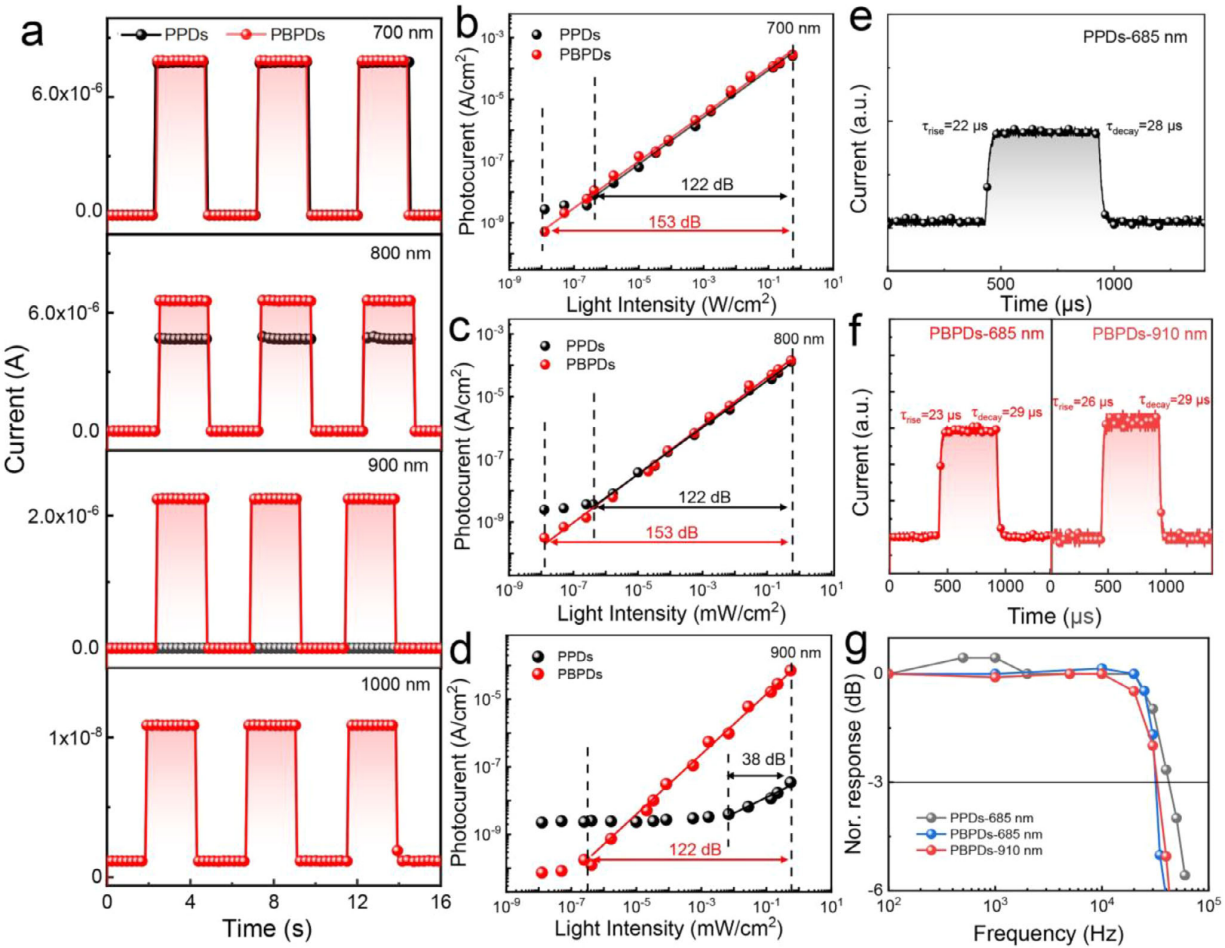
a) Current–Time responses of PPDs and PBPDs under light illuminations of different wavelength with the same power density of 20 mW cm^−2^ for the photocurrent compression of PPDs and PBPDs at different wavelengths. b–d) *LDR* or photosensitivity linearity of PPDs and PBPDs at 700, 800, and 900 nm (*V_bias_* = 0 V). e) The response time of the PPDs at 685 nm, and f) PBPDs at 685 and 910 nm. g) Frequency response of PPDs and PBPDs.

The current–time curve further demonstrates that the light response is extended to the NIR band. As shown in **Figure** **3**a, PBPDs exhibit an on‐off ratio above the 10^4^ under 700, 800, and 900 nm of light sources. On the other hand, PPDs only have a considerable response in the visible light range, and barely respond to light sources higher than 900 nm. Linear dynamic range (*LDR*) quoted in dB is a direct parameter to evaluate the light intensity detection range of the device. It is estimated by collecting the linear relationship of photocurrent and light intensity according to the following equation:

(5) $$\mathrm{LDR}=20\log\frac{J_{\max}}{J_{\min}}$$

where *J_max_* and *J_min_* denote the linear part of the maximum and minimum of detectable light intensity, respectively. The photocurrent versus the light intensity plots of PPDs and PBPDs are shown in Figure 3b–d. It is estimated that PBPDs have high *LDR* of 153 dB both at 700 and 800 nm, which is higher than those of PPDs at 122 dB and comparable to those of commercial PDs based on inorganic semiconductor materials.^[^ ^46^ ^]^ To note, the PBPDs could also exhibit an increased *LDR* of 122 dB as the incident NIR light expands to 900 nm, indicating that the efficient NIR light harvest is benefiting from the favorable double cascading charge transfer paths as shown in Figure 2b.

The time response of PPDs and PBPDs are conducted by the transient photocurrent (TPC) method as shown in Figure 3e,f. The rise time of PPDs to the 685 nm laser is 22 µs with a recovery time of 28 µs. The PBPDs exhibit the rise/decay time of 23/29 and 26/29 µs to the 685 and 910 nm light source, respectively. Surprisingly, the incorporated organic layer doesn't affect the response speed. The −3 dB bandwidth (*f*_−3 dB_) is measured by using the normalized response intensity versus the incident light frequency (Figure 3g). The −3 dB cutoff frequencies of PBPDs are determined as 3.3 × 10^4^ and 3.1 × 10^4^ Hz at 685 and 910 nm which are very close to PPDs of 4.2 × 10^4^ Hz at 685 nm. This fast response time in the microsecond level and the wide spectrum response make these PDs promising for applications in highly sensitive light detection and visible optical communication.

The coating of a hydrophobic organic film could also inhibit the penetration of humidity and improve operation stability. The contact angle test (Figure S3a–c, Supporting Information) shows that the water contact angle on perovskite surface is 56.6°. After the spin‐coating of the PC_61_BM layer, the contact angle increases to 90.4° and after the coating of the BHJ layer, the contact angle further increases to 103.4°. As a result, PBPDs with the BHJ layer have better air stability up to 100 h, while PPDs decays rapidly in air for 48 h under a relative humidity of 60% (Figure S3d, Supporting Information).

**Figure 4:**
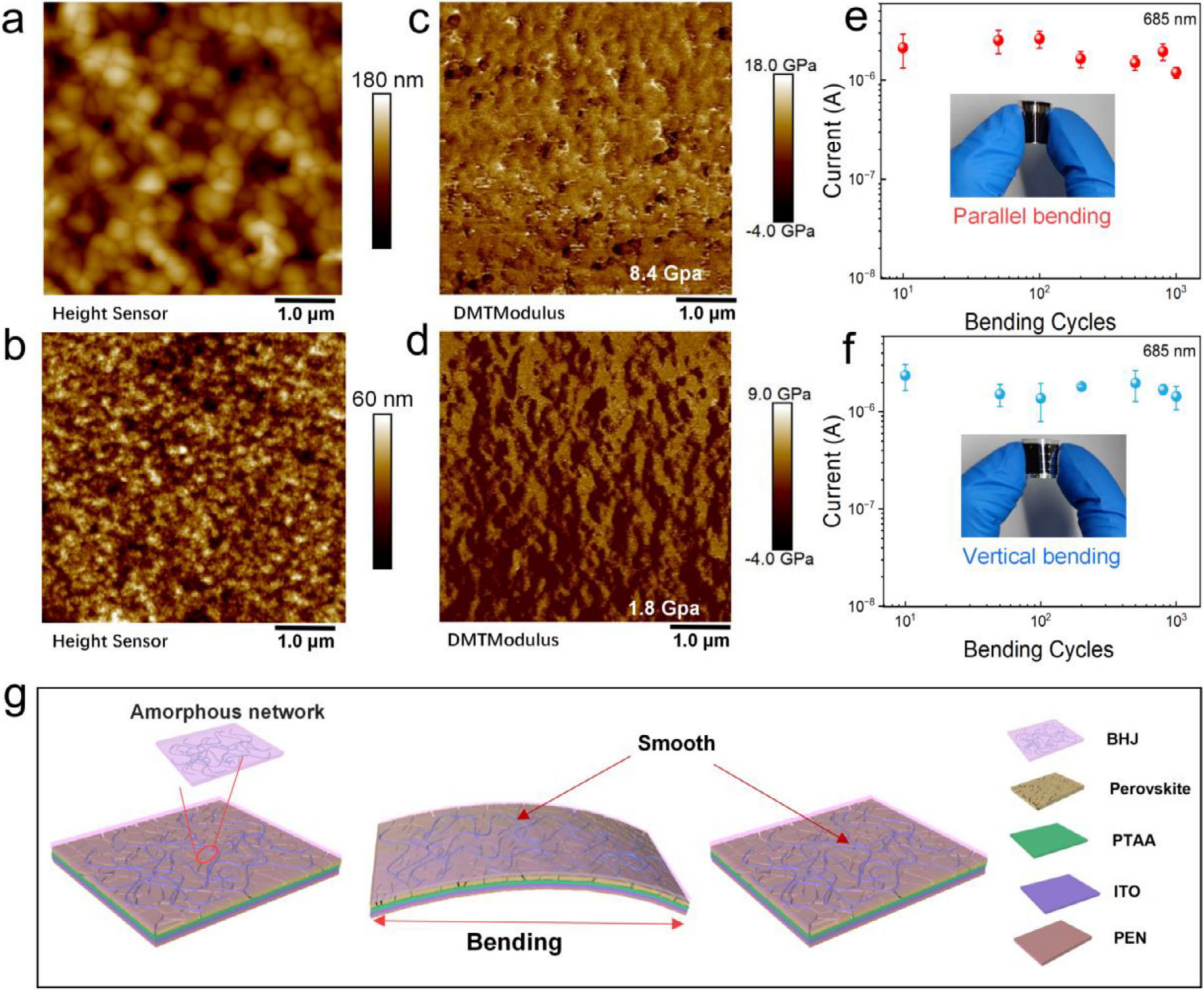
AFM phase and height images for perovskite film a) and BHJ b) on the top. Young's modulus of perovskite film with PC_61_BM c) and BHJ film d). e,f) Photocurrent variations of flexible PBPDs versus bending cycles with parallel and vertical direction (*r* is fixed at 5 mm). The error bars represent the standard deviation derived from measurements on multiple devices (*n* ≥ 3). g) The possible mechanism of action by the BHJ layer e,f).

Perovskite devices could be processed by solution methods but they can be easily damaged by tensile or compressive stresses due to their mechanical rigidity. For example, cracks in the perovskite active layer or transport layer could cause unnecessary device leakage current and short circuits. In contrast, polymer‐based OPDs usually have excellent bending properties since polymers with long‐chain could form amorphous network to enhance molecular interactions for better elastic deformation. Small‐molecule BTP‐4F and PC_61_BM tend to form crystallization structures.^[^ ^53^, ^54^ ^]^ The differential stress release mechanisms observed under cyclic stretching conditions manifest through distinct molecular‐to‐mesoscale processes that define material performance. The morphology and roughness of the film are illustrated in the AFM images presented in **Figures** **4**a,b and S4 (Supporting Information). The perovskite film exhibits a rugged surface characterized by various sizes of crystal grains, with a roughness measuring 20.4 nm. Upon deposition of the BHJ layer, the surface roughness diminishes to 2.1 nm. Furthermore, long nanofibers are entangled within the pristine D18 polymer film, whereas pure BTP‐4F and PC_61_BM films appear amorphous. Therefore, the BHJ layer has the well‐formed D18 nanofiber embedded around the amorphous BTP‐4F and PC_61_BM mixture, potentially indicating better bending capability. The Young's modulus of perovskite films with PC_61_BM and BHJ is depicted in Figure 4c,d. Nanomechanical mapping reveals significant spatial heterogeneity in local stiffness across the perovskite thin films. Specifically, the perovskite with a PC_61_BM film exhibits a modulus of 8.4 GPa, whereas the perovskite with a BHJ film displays a lower modulus of 1.8 GPa. This finding suggests that the integrated structure of perovskite/BHJ possesses improved bending stability.

The top‐view SEM images in Figure S5 (Supporting Information) further reveal the morphological changes in perovskite films with PC_61_BM and BHJ during the bending process. Following the 1000‐cycle bending test, cracks become evident in the perovskite film with PC_61_BM, whereas no noticeable cracks are observed in the perovskite film with BHJ. This result suggests that the molecular backbone of D18 polymer could effectively reduce cracking during the continuous bending process. As revealed by the AFM image, the microstructure of the organic films displays a certain level of orientation in the distribution of chain‐like polymers. However, it is expected that the skeleton structure of the device should exhibit a uniform distribution without any preferential orientation. To assess the bending performance of the device, flexible prototypes were deposited on a polyethylene naphthalate (PEN) substrate coated with indium tin oxide (ITO), and subjected to a bending test of 1000 cycles with a curvature radius of 5 µm in three different directions (parallel, vertical, and diagonal). Results depicted in Figure 4e,f demonstrates that PBPDs upon light excitation shows remarkable stability in the bending test, with only a slight reduction in the photocurrent. This suggests that the randomly distributed long‐chain D18 polymer in the organic BHJ layer may improve the device's omnidirectional bending stability. Conversely, the device based on PPDs undergoes severe damage as shown in Figure S6 (Supporting Information). These findings further validate the effectiveness of BHJ layer embedded in chain‐like polymer network in improving the bending performance of the device. Figure 4g shows the schematic diagram of the improvement in bending by the organic framework structure as the organic polymer network in the BHJ provides the elastic support to release the strain during the bending process.

**Figure 5:**
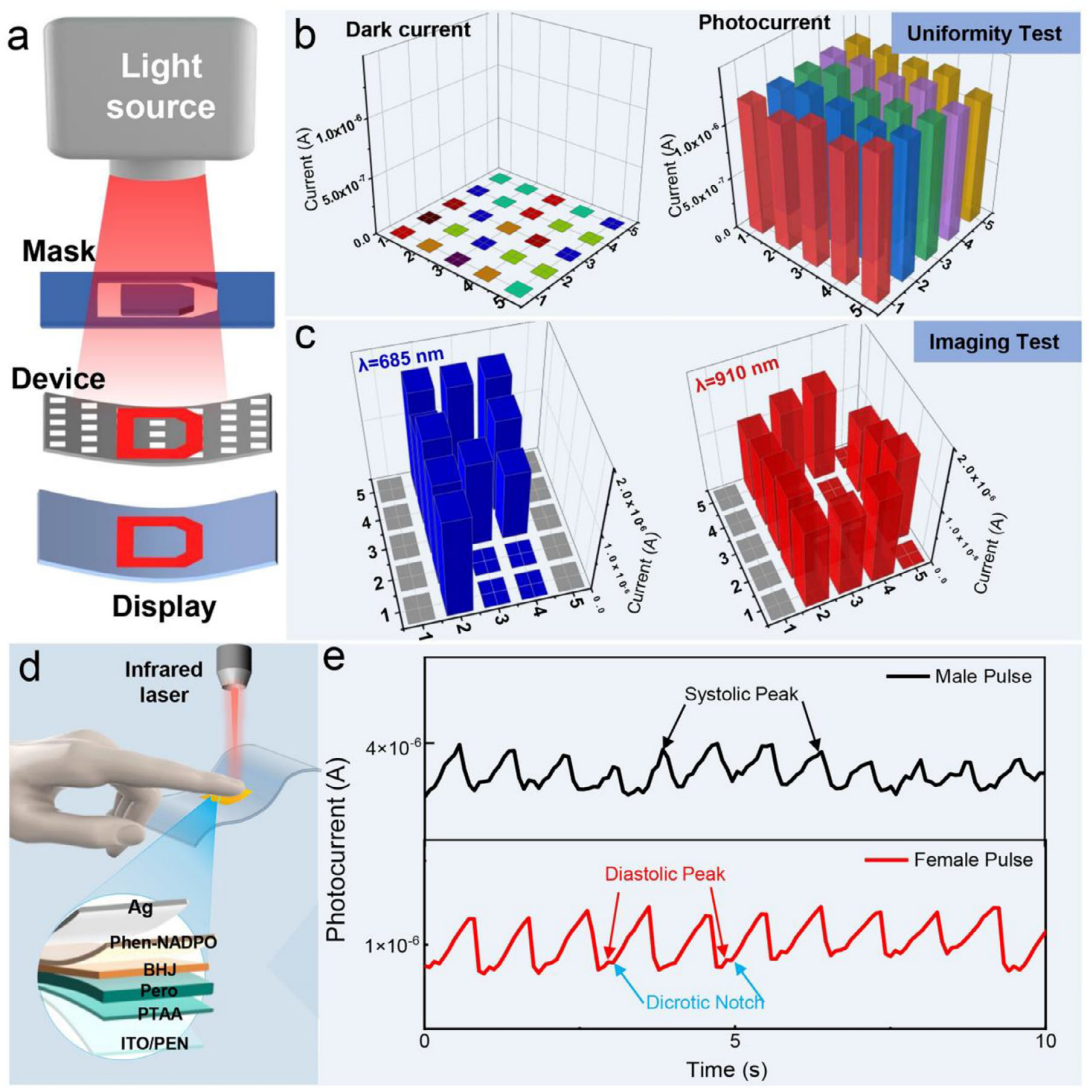
a) Working process diagram of flexible image arrays and NIR photoplethysmography based on PBPDs. b) The photocurrent of the PBPDs array (25 pixels with a typical pixel size of 1 × 1 mm) under dark and 905 nm light illumination, respectively. c) Sensing results of PBPDs in the visible (685 nm) and NIR light (910 nm) by using the “P‐” and “D‐” letter masks, respectively. d)The schematic of detecting NIR photoplethysmography by using flexible PBPDs. The optical power density of the 910 nm NIR light incident on the skin for heart rate monitoring was maintained at ≈3.5 mW cm^−2^. e) Pulse signal of male and female volunteers measured by the flexible devices under a NIR light of 910 nm.

By extending the detection range to NIR with good flexibility, PBPDs have the great potential for applications in visible‐NIR imaging and physiological signal monitoring for wearable devices. An array with 25 pixels of PBPDs is fabricated and a schematic of the measurement setup is shown in **Figure** **5**a and Figure S7 (Supporting Information). Photoelectric signals generated by each pixel are transmitted and addressed through the two sets of intersecting bottom ITO and top Ag electrodes. When either the visible light (685 nm) or NIR light (910 nm) passes through a pre‐designed mask or human finger, photoelectric signals generated by each pixel is transmitted through two intersecting electrodes. Figure 5b shows the current distribution of the PBPDs without (left) or with light illumination (right), demonstrating the low standard deviation of (1.04 ± 0.37) × 10^−10^ (in dark) and (1.25 ± 0.04) × 10^−6^ A (under illumination), respectively. Therefore, the as‐obtained 25 pixels of PBPDs have exhibited a satisfying uniformity for the photoelectric sensitivity. By placing a mask with either the “P” or “D” letter between the laser and the device, the resulting images were clearly recognizable (Figure 5c). The flexible PBPDs also enable a better conformal fit with the body for better vital sign measurements in the NIR mode. A photoplethysmography is performed to monitor the pulse signals of heart beating (Figure 5d). The periodic changes in blood volume could cause the variations of transmitted light, as a result, the photoelectric signals of PBPD with very high sensitivity are simultaneously generated and recorded for heart rate measurement accordingly. Figure 5e shows the typical systolic and diastolic peaks of heart rate with 12 and 11 cardiac cycles in 20 s for a male and female volunteer, respectively and the heart rate per minute is 72 and 66, respectively.

**Figure 6:**
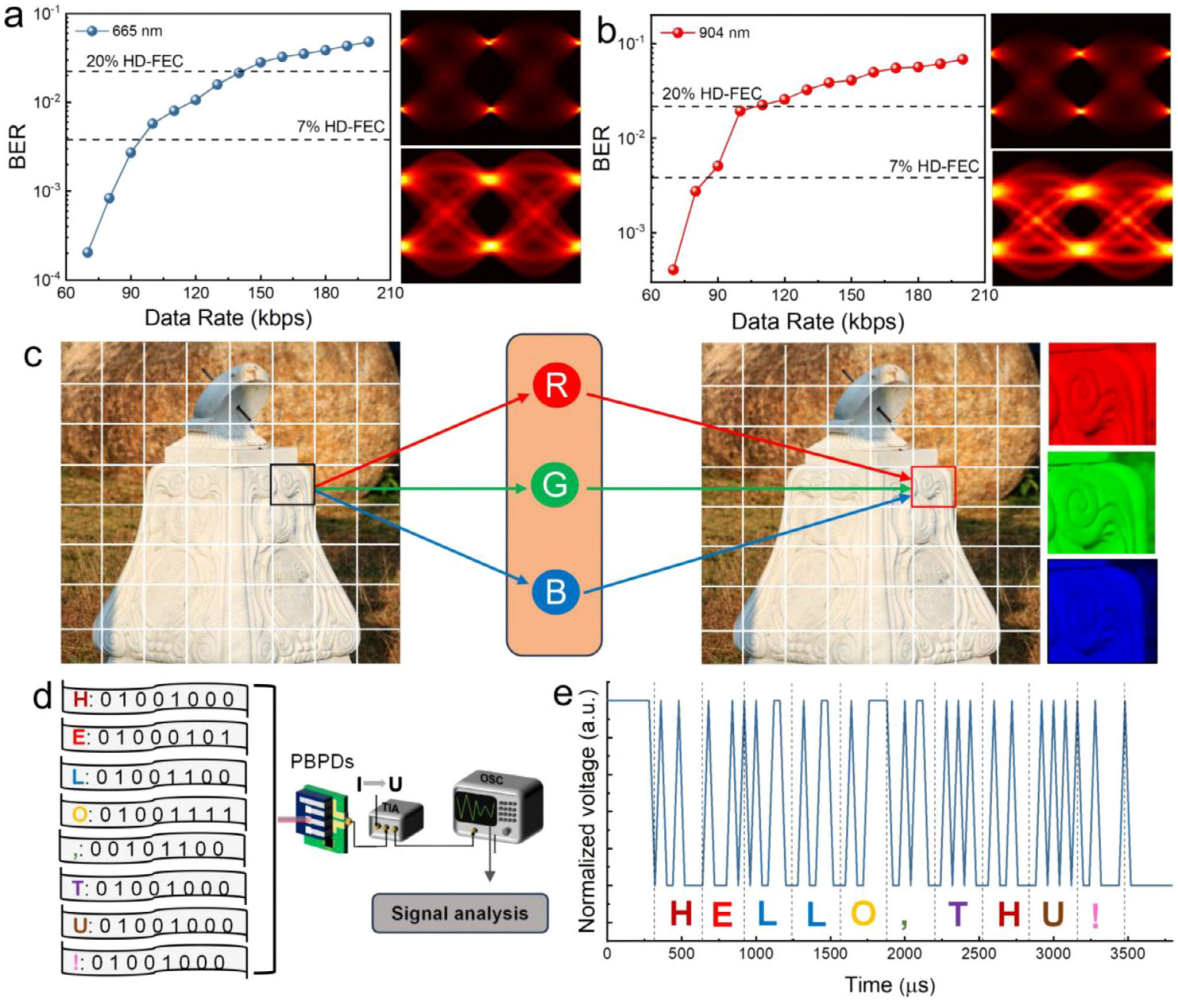
a) Bit error rate test utilizing PBPDs Detector with light sources at 665 nm a) and 904 nm b) LED light. c) image transfer using PBPDs with 904 nm LED Light. d,e) illustration of the word transmission process and transmission data diagram.

The performance of PBPDs in the visible and NIR spectrums has been outstanding, prompting further investigations into their application in optical communication systems. Utilizing a 665 nm communication link, the PBPDs system demonstrated optimal data transmission capabilities, achieving a maximum rate of 90 kbps (kilobits per second) within a 7% Forward Error Correction (HD‐FEC) threshold, corresponding to a Bit Error Rate (BER) of 3.8 × 10^−3^. At a higher tolerance of 20% HD‐FEC, the data rate increased to 140 kbps (as illustrated in **Figure** **6**a). Meanwhile, the 904 nm link showed that the system could handle data rates of 140 and 110 kbps under 7% and 20% HD‐FEC thresholds, respectively. Extending the scope of our analysis, eye diagram assessments were conducted at transmission rates of 80 and 180 kbps (Figure 6b). At the lower rate, the eye diagrams exhibited pronounced clarity, indicating the robustness of signal integrity. In contrast, the eye diagrams at 180 kbps displayed a constricted appearance, implying that the photodetector might be operating beyond its optimal performance capacity. This phenomenon provides critical insights into the bandwidth limitations of the device and sets a precedent for subsequent enhancement strategies. Furthermore, the PBPDs demonstrated efficient transmission of clear pictural information under a 904 nm LED light source, as depicted in Figure 6c. Additionally, character data were successfully encoded at a rate of 25 kbps, with each character represented by a sequence of eight binary digits, providing further evidence of the photodetector's ability to handle complex data transmission, as seen in Figure 6d,e. These results underscore the potential of PBPDs in advanced optical communication technologies, highlighting their capacity for high‐fidelity data transmission over varied spectral ranges.

## 3 Conclusion

This study has successfully elucidated the multifunction roles of the PC_61_BM:D18:BTP‐4F ternary organic BHJ in improving the performance of perovskite based photodetectors. Key enhancements include the extension of the photoresponse from 800 to 950 nm, enabling the broadband responsivity across the visible‐to‐NIR spectrum. The formation of the idealized heterojunction with perovskite layer has significantly suppressed the leakage current, realizing the dark current (*J*_D_) of 10^−11^ A cm^−2^ and noise current density of 10^−14^ A Hz^−1/2^. Additionally, the incorporation of the PC_61_BM within the BHJ layer facilitates dual electron transport path ways alongside BTP‐4F, resulting in fast response and enhanced charge collection efficiency. The structural configuration of the BHJ layer, featuring a chain‐like interpenetrating polymer network, significantly mitigates mechanical strain during bending, thereby enhancing device flexibility. The hydrophobic surface network of the BHJ also acts as a barrier against ambient moisture, preserving the integrity of the perovskite layer and thus fortifying operational stability. These enhancements enable perovskite‐based broadband photodetector (PBPDs) to achieve a wide linear response range exceeding 122 dB, coupled with self‐powered high detectivities of 10^12^ *Jones* at 900 nm without any external bias voltage. The 25‐pixel (5 × 5) flexible PD array upholds complete functional stability in ambient environments, demonstrating consistent spatial uniformity under both dark and illuminated conditions. In terms of optical communication, the photodetector's low bit error rate, spanning visible (λ = 665 nm) and NIR (λ = 904 nm) spectra, and its capacity to transmit images and text at 90 and 140 kbps respectively. Its practical applications, such as high‐fidelity NIR imaging and non‐contact heart‐rate monitoring, have been successfully validated, highlighting its optical excellence and functional capabilities. While the response time in the microsecond range and the resulting data rates are sufficient for the demonstrated applications such as wearable monitoring and medium‐speed communication, the device speed is primarily limited by circuit‐related parameters such as resistance and capacitance (RC) time constant. Future work to minimize the active area and optimize layer thicknesses and mobilities is expected to push the bandwidth into the megahertz range, enabling gigabit‐rate data transmission for advanced optical communication systems.

In conclusion, we have demonstrated a high‐performance flexible photodetector through the integration of a CsFAPbI_3_ perovskite with an optimized ternary organic bulk‐heterojunction (D18:BTP‐4F:PC_61_BM). This unique combination, featuring cascading energy levels and dual charge transport pathways, enables a breakthrough in noise suppression. The integrated perovskite/organic device not only presents a pioneering approach but also substantiates the fundamental operating principles required for achieving broadband detection, self‐powered functionality, and air‐stable flexibility, all of which are imperative for the advancement of wearable electronic technologies and optical communications.

## Conflict of Interest

The authors declare no conflict of interest.

## Supporting information



Supporting Information

## Data Availability

Research data are not shared.

## References

[1] F. Wang , X. Zou , M. Xu , H. Wang , H. Wang , H. Guo , J. Guo , P. Wang , M. Peng , Z. Wang , Y. Wang , J. Miao , F. Chen , J. Wang , X. Chen , A. Pan , C. Shan , L. Liao , W. Hu , Adv. Sci. 2021, 8, 2100569. 10.1002/advs.202100569 PMC8292906 34032025

[2] Z. J. Lan , Y. S. Lau , L. F. Cai , J. Y. Han , C. W. Suen , F. R. Zhu , Laser Photonics Rev. 2022, 16, 2100602.

[3] W. Tian , H. P. Zhou , L. Li , Small 2017, 13, 1702107.

[4] C. Bao , Z. Chen , Y. Fang , H. Wei , Y. Deng , X. Xiao , L. Li , J. Huang , Adv. Mater. 2017, 29, 1703209. 10.1002/adma.201703209 28846818

[5] H. Wu , Y. Sun , D. Lin , R. Zhang , C. Zhang , W. Pan , Adv. Mater. 2009, 21, 227.

[6] W. Yang , K. Hu , F. Teng , J. Weng , Y. Zhang , X. Fang , Nano Lett. 2018, 18, 4697. 30052044 10.1021/acs.nanolett.8b00988

[7] H. Tan , C. Fan , L. Ma , X. Zhang , P. Fan , Y. Yang , W. Hu , H. Zhou , X. Zhuang , X. Zhu , A. Pan , Nano‐micro Lett. 2016, 8, 29. 10.1007/s40820-015-0058-0 PMC6223916 30464991

[8] X. Zhang , Y. Huang , X. Duan , X. Fan , Q. Wang , X. Zhang , B. Shen , W. Wang , X. Ren , Opt. Commun. 2012, 285, 4338.

[9] A. Rogalski , J. Antoszewski , L. Faraone , J. Appl. Phys. 2009, 105, 091101.

[10] H. Wang , D. H. Kim , Chem. Soc. Rev. 2017, 46, 5204. 28795697 10.1039/c6cs00896h

[11] J. Li , G. Zhang , Z. Zhang , J. Li , Z. Uddin , Y. Zheng , Y. Shao , Y. Yuan , B. Yang , ACS Appl. Mater. Interfaces 2021, 13, 56358. 34788529 10.1021/acsami.1c19323

[12] L. Zheng , T. Zhu , W. Xu , J. Zheng , L. Liu , ACS Sustainable Chem. Eng. 2018, 6, 12055.

[13] C. K. Liu , Q. Tai , N. Wang , G. Tang , H. L. Loi , F. Yan , Adv. Sci. 2019, 6, 1900751. 10.1002/advs.201900751 PMC6724360 31508281

[14] L. Dou , Y. Yang , J. You , Z. Hong , W.‐H. Chang , G. Li , Y. Yang , Nat. Commun. 2014, 5, 5404. 25410021 10.1038/ncomms6404

[15] J. Zhou , J. Huang , Adv. Sci. 2018, 5, 1700256. 10.1002/advs.201700256 PMC5770665 29375959

[16] W. Xu , Y. Guo , X. Zhang , L. Zheng , T. Zhu , D. Zhao , W. Hu , X. Gong , Adv. Funct. Mater. 2018, 28, 1705541.

[17] S. Tan , T. Huang , I. Yavuz , R. Wang , T. W. Yoon , M. Xu , Q. Xing , K. Park , D.‐K. Lee , C.‐H. Chen , R. Zheng , T. Yoon , Y. Zhao , H.‐C. Wang , D. Meng , J. Xue , Y. J. Song , X. Pan , N.‐G. Park , J.‐W. Lee , Y. Yang , Nature 2022, 605, 268. 35292753 10.1038/s41586-022-04604-5

[18] Y. Zhang , Y. Ma , Y. Wang , X. Zhang , C. Zuo , L. Shen , L. Ding , Adv. Mater. 2021, 33, 2006691. 10.1002/adma.202006691 34028107

[19] W. Z. Xu , Y. Gao , M. He , S. Y. Chen , H. Y. Fu , G. D. Wei , Nano Res. 2023, 16, 481.

[20] M. I. Saidaminov , V. Adinolfi , R. Comin , A. L. Abdelhady , W. Peng , I. Dursun , M. Yuan , S. Hoogland , E. H. Sargent , O. M. Bakr , Nat. Commun. 2015, 6, 8724. 26548941 10.1038/ncomms9724 PMC4667636

[21] P. C. Y. Chow , T. Someya , Adv. Mater. 2020, 32, 1902045. 10.1002/adma.201902045 31373081

[22] Y. Song , Z. Zhong , P. He , G. Yu , Q. Xue , L. Lan , F. Huang , Adv. Mater. 2022, 34, 2201827. 10.1002/adma.202201827 35561337

[23] L. Shen , Y. Lin , C. Bao , Y. Bai , Y. Deng , M. Wang , T. Li , Y. Lu , A. Gruverman , W. Li , J. Huang , Mater. Horiz. 2017, 4, 242.

[24] C. Li , H. Wang , F. Wang , T. Li , M. Xu , H. Wang , Z. Wang , X. Zhan , W. Hu , L. Shen , Light Sci. Appl. 2020, 9, 31. 32194945 10.1038/s41377-020-0264-5 PMC7054320

[25] Y.u Zhang , Z. Qin , W. Nie , Y. Li , X. Huo , D. Song , B.o Qiao , Z. Xu , S. Wageh , A. Al‐Ghamdi , S. Zhao , Adv. Opt. Mater. 2022, 10, 2200648.

[26] Y. X. Xia , L. E. Aguirre , X. F. Xu , O. Inganäs , Adv. Electron. Mater. 2020, 6, 1901017.

[27] P. Szuromi , Science 2020, 370, 677.

[28] Y. S. Liu , Y. S. Chen , Adv. Mater. 2020, 32, 1805843. 10.1002/adma.201805843 30773710

[29] Y. Gao , W. Xu , S.‐W. Zhang , T. Fan , M. Zhang , A. Ran , X. Zhang , F. Kang , G. Wei , Small 2022, 18, 2106083. 10.1002/smll.202106083 35106905

[30] Y. Gao , C. Zhao , K. Pu , M. He , W. Cai , M.‐C. Tang , F. Kang , H.‐L. Yip , G. Wei , Sci. Bull. 2022, 67, 1982. 10.1016/j.scib.2022.09.007 36546208

[31] J. Yuan , Y. Zhang , L. Zhou , G. Zhang , H.‐L. Yip , T.‐K. Lau , X. Lu , C. Zhu , H. Peng , P. A. Johnson , M. Leclerc , Y. Cao , J. Ulanski , Y. Li , Y. Zou , Joule 2019, 3, 1140.

[32] Q. Liu , Y. Jiang , K. Jin , J. Qin , J. Xu , W. Li , J. Xiong , J. Liu , Z. Xiao , K. Sun , S. Yang , X. Zhang , L. Ding , Sci. Bull. 2020, 65, 272. 10.1016/j.scib.2020.01.001 36659090

[33] R. Xu , Z. Wang , W. Xu , X. Xu , J. Wang , Z. Hu , Y.u Li , G. Wang , W. Cai , S. Zheng , G. Wei , F. Huang , S. Yang , Sol. RRL 2021, 5, 2100236.

[34] J. Wei , F. Guo , B. Liu , X. Sun , X.i Wang , Z. Yang , K. Xu , M. Lei , Y. Zhao , D. Xu , Adv. Energy Mater. 2019, 9, 1901620.

[35] Z. Zhu , Q. Xue , H. He , K. Jiang , Z. Hu , Y. Bai , T. Zhang , S. Xiao , K. Gundogdu , B. R. Gautam , H. Ade , F. Huang , K. S. Wong , H.‐L. Yip , S. Yang , H. Yan , Adv. Sci. 2016, 3, 1500353. 10.1002/advs.201500353 PMC5039980 27711265

[36] J. Wu , H. Cha , T. Du , Y. Dong , W. Xu , C.‐T. Lin , J. R. Durrant , Adv. Mater. 2022, 34, 2101833. 34773315 10.1002/adma.202101833 PMC11469080

[37] Q. Han , S.‐H. Bae , P. Sun , Y.‐T. Hsieh , Y. M. Yang , Y. S. Rim , H. Zhao , Q. I Chen , W. Shi , G. Li , Y. Yang , Adv. Mater. 2016, 28, 2253. 26790006 10.1002/adma.201505002

[38] M. Hou , H. Zhang , Z.e Wang , Y. Xia , Y. Chen , W. Huang , ACS Appl. Mater. Interfaces 2018, 10, 30607. 30118201 10.1021/acsami.8b10332

[39] Y. Xie , Z. Ding , T. Lu , Y. Shao , Z. Yang , S. Jiang , Chem. Eng. J. 2025, 519, 164916.

[40] M. Liu , Q. Yao , S. Li , Y. Qin , S. Y. Jeong , Y. Ma , L. Shen , X. Ma , K. Yang , G. Yuan , H. Y. Woo , F. Zhang , Adv. Optical Mater. 2024, 12, 2303216.

[41] W. A. Laban , L. Etgar , Energy Environ. Sci. 2013, 6, 3249.

[42] W. Xu , Y.u Gao , K. Qian , B. Wang , R. Xu , M. He , T. Li , G. Xing , S. Yang , G. Wei , J. Mater. Chem. C 2022, 10, 9391.

[43] P. Liu , Y. Liu , S. Zhang , J. Li , C. Wang , C. Zhao , P. Nie , Y. Dong , X. Zhang , S. Zhao , G. Wei , Adv. Opt. Mater. 2020, 8, 2001072.

[44] A. Morteza Najarian , M. Vafaie , A. Johnston , T. Zhu , M. Wei , M. I. Saidaminov , Y. Hou , S. Hoogland , F. Pelayo García de Arquer , E. H. Sargent , Nat. Electron. 2022, 5, 511.

[45] J. Wang , X. Xu , S. Xiao , Y. Li , W. Qian , J. Yu , K. Zhang , S. Yang , Adv. Opt. Mater. 2021, 9, 2100517.

[46] X. Gong , M. Tong , Y. Xia , W. Cai , J. S. Moon , Y. Cao , G. Yu , C.‐L. Shieh , B. Nilsson , A. J. Heeger , Science 2009, 325, 1665. 19679770 10.1126/science.1176706

[47] M. Zhang , F. Zhang , Y. Wang , L. Zhu , Y. Hu , Z. Lou , Y. Hou , F. Teng , Sci. Rep. 2018, 8, 11157. 30042485 10.1038/s41598-018-29147-6 PMC6057967

[48] Z. Li , H. Li , K.e Jiang , D. Ding , J. Li , C. Ma , S. Jiang , Y.e Wang , T. D. Anthopoulos , Y. Shi , ACS Appl. Mater. Interfaces 2019, 11, 40204. 31599148 10.1021/acsami.9b11835

[49] Y. Wei , H. Chen , T. Liu , S. Wang , Y. Jiang , Y.u Song , J. Zhang , X. Zhang , G. Lu , F. Huang , Z. Wei , H. Huang , Adv. Funct. Mater. 2021, 31, 2106326.

[50] J. Huang , J. Lee , J. Vollbrecht , V. V. Brus , A. L. Dixon , D. X. Cao , Z. Zhu , Z. Du , H. Wang , K. Cho , G. C. Bazan , T.‐Q. Nguyen , Adv. Mater. 2020, 32, 1906027. 10.1002/adma.201906027 31714629

[51] J. Liu , J. Jiang , S. Wang , T. Li , X. Jing , Y. Liu , Y. Wang , H. Wen , M. Yao , X. Zhan , L. Shen , Small 2021, 17, 2101316. 10.1002/smll.202101316 34114339

[52] M. Babics , H. Bristow , W. Zhang , A. Wadsworth , M. Neophytou , N. Gasparini , I. McCulloch , J. Mater. Chem. C 2021, 9, 2375.

[53] S. Wu , Z. Li , J. Zhang , X. Wu , X. Deng , Y. Liu , J. Zhou , C. Zhi , X. Yu , W. C. H. Choy , Z. Zhu , A. K.‐Y. Jen , Adv. Mater. 2021, 33, 2105539. 10.1002/adma.202105539 34601764

[54] N. J. Zhou , A. Facchetti , Mater. Today 2018, 21, 377.

