# Supplementary material for: Organic Bulk‐Heterojunction‐Integrated Flexible Perovskite Photodetection Arrays for High‐Speed Broadband Optical Communication
Source: Adv Sci (Weinh). 2025 Dec 8;13(9):e09546. doi: 10.1002/advs.202509546 (PMC12904039; doi:10.1002/advs.202509546)
Supplement: Supplementary file 1 — Supporting Information [file ADVS-13-e09546-s001.docx]

**Organic Bulk-Heterojunction-Integrated Flexible Perovskite Photodetection Arrays for High-Speed Broadband Optical Communication**

***Materials***

The Poly (triaryl amine) (PTAA, 99.8%), PbI_2_, Formamidinium iodide (FAI, 99.9%), [6,6]-Phenyl C61 butyric acid methyl ester (PC_61_BM, 99.7%) were bought from Xi’an Polymer Light Technology Corp. (China). CsI (99.8%, Alfa Aesar). Phen-NADPO (98%, Purui Materials). Toluene (TL, Energy Chemical, 99.98%); Chloroform (CF, Energy Chemical, 99.8%). Dimethylformamide (DMF, 99.8%), dimethyl sulfoxide (DMSO, 99.9%), chlorobenzene (CB, 99.99%) were obtained from Sigma-Aldrich; Isopropanol (IPA, Macklin, 99.8%); D18 (98%,1 material inc.); BTP-4F (99.9%, 1 material inc.); Ethanol (99.7%,); Acetone (99.8%) were purchased from aladdin;

***Preparation of the precursor***

The 1.1 M Cs_0.15_FA_0.85_PbI_3_ solution was prepared by dissolving 21.93 mg CsI, 160.79 mg FAI, and 507.2 mg PbI_2_ in 1 mL of a mixed solution with a volume ratio of DMF:DMSO = 4:1. The perovskite precursor solution was filtered through a 0.22 μm PTFE syringe filter (Jinteng, China) prior to spin-coating. The ternary organic solution was prepared by mixing PC_61_BM, D18, and BTP-4F in a mass ratio of 4.8:1:1.6 (with a total concentration of 15 mg/mL) in CF. Additionally, a solution of 20 mg/mL PC_61_BM was also dissolved in chloroform.

***Device Fabrication***

The ITO glass substrates were sequentially rinsed with ethanol, deionized water, and acetone through ultrasonic treatment (20 minutes per solvent cycle), followed by thermal annealing in a precision-controlled drying oven to remove residual solvents. A 120-second oxygen plasma treatment was applied to generate a hydrophilic surface. The PTAA hole transport layer was fabricated by spin-coating a 2 mg/mL toluene-based solution onto preprocessed ITO substrates (5000 rpm, 20 s), followed by thermal annealing at 110°C for 10 min in a nitrogen-atmosphere glovebox. The CsFAPbI_3_ active layer was deposited via a two-step spin-coating process (1000 rpm for 8 s followed by 5000 rpm for 30 s) on PTAA-modified substrates. A 100 μL chlorobenzene (CB) anti-solvent treatment was applied during the second spinning stage. Thermal treatment at 100°C for 30 min completed the crystallization process. A PC_61_BM:D18:BTP-4F ternary blend or PC_61_BM single-component solution was applied by spin-coating (2000 rpm, 30 s). Subsequently, a Phen-NADPO interfacial layer (0.5 mg/mL in isopropanol) was deposited at 2000 rpm for 30 seconds. Finally, thermal evaporation of 100 nm silver electrodes under high vacuum conditions (4.0×10^-4^ Pa base pressure). Flexible devices employ similar thin film deposition methods, utilizing PEN/ITO substrates.

***Device measurement and Characterization***

*I*-*V* and *I*-*t* tests were carried out by a home-build system consisting of optical (SC-pro and AOTF-PRO (OYSL, produce 430-1450 nm light)) and electrical parts (Probe and Keithley 2600B). The semiconductor analyzing system and probe station were used to measure the current-time curves (Keithley 4200A-SCS, USA, Lake Shore, USA). The frequency response system was employed to quantify the response speed. Surface topography analysis was performed using a surface profilometer (MDTC-EQ-M16-01, Bruker Dimension Icon) and field-emission scanning electron microscope (SU8010, HITACHI). Optical absorption properties were characterized through UV-vis spectroscopy (HP 8453 spectrometer). Wettability assessment employed a Kruss DSA30 contact angle analyzer; The noise current density characterization was performed using an optically isolated and electrically shielded probe station, which was serially interfaced with an SR830 lock-in amplifier (Stanford Research). DC bias was supplied via battery power source. The noise current expressed in A Hz^1/2^ was quantified through a lock-in amplifier with integration time adjustments. System validation was conducted employing a pre-characterized silicon photodetector (commercial grade) with quantified Noise-Equivalent Power (NEP) parameters. All the research participants including for experiments with as-obtained perovskite/organic photodetectors to test the heart rate through illuminating light on touchless fingers have been described and consented.

***Bit error rate test:*** We utilized a conventional Orthogonal Frequency Division Multiplexing (OFDM) method customized for evaluating Intensity Modulation/Direct Detection (IM/DD) systems. A pseudorandom bit sequence was synthesized using MATLAB, which subsequently underwent mapping into Quadrature Amplitude Modulated (QAM) symbols. These serial symbols were restructured into parallel streams through Serial-to-Parallel (S/P) conversion, arranged into Hermitian symmetry to facilitate the Inverse Fast Fourier Transform (IFFT). Post-IFFT, a Cyclic Prefix (CP) was appended to each frame to mitigate intersymbol interference, followed by a Parallel-to-Serial (P/S) conversion to formulate the OFDM signal. This signal was then converted into an analog waveform via an Arbitrary Waveform Generator (AWG), which in turn modulated the light sources — specifically, a 665-nm Laser Diode (LD) (DV0680M, Deray) and a 904-nm LD (L904P010, Thorlabs). An aspheric lens collimated the emitted light beams, which were subsequently transmitted through the testing medium. At the receiving end, an aspheric lens concentrated the incoming optical signals onto the custom-designed Photodetector (PD) module. The received electrical signal was captured by an oscilloscope and subjected to standard digital signal processing techniques, including synchronization, CP removal, Fast Fourier Transform (FFT) application, and channel equalization, to reconstruct the original transmitted data stream. The resulting data was then demodulated, translating the QAM symbols back into a bitstream for Bit-Error-Rate (BER) performance assessment.

***Picture transmission principle:*** In this optical wireless communication demonstration, each pixel within a monochrome image or a color channel of an RGB image is quantized into 256 discrete levels, therefore it stores 8 bits information. Consequently, a square image whose side length equals 600 pixels can be represented by 864, 000 binary digits. For convenience of processing, they are stored in a sequence called “Plaintext Sequence”, rather than in a matrix.

Encryption is performed using a codebook which is securely stored locally at both the transmitter and receiver and contains several seeds for Lorenz chaotic systems. A seed is selected to generate a corresponding Lorenz chaotic sequence, aligning in length with the “Plaintext Sequence”. Then a bitwise exclusive OR (XOR) operation between the two sequences yields an encrypted “Ciphertext Sequence” of equivalent length. A pre-defined sequence for training the receiver's Least Mean Squares (LMS) equalizer is appended to the “Ciphertext Sequence”.

The next phase involves an unequally-spaced PAM4 scheme, where the constellation distance ratio equals 2. Here, every pair of binary digits is grouped and mapped to one of four potential signal levels—0, 0.25, 0.75, 1—corresponding to the binary codes 00, 01, 11, and 10, respectively. This modulation reduces the digital sequence length by half, as each pair of digits is represented by a single new value. A synchronization sequence is added to the modulated sequence for ease of synchronization and identification by the receiver.

Upon reception, synchronization, and identification, the receiver first extracts the “Ciphertext Sequence” and training sequence. The latter is utilized to optimize the equalizer for subsequent equalization. The received and equalized “Ciphertext Sequence” is undergone the normalization to ensure an average power of 1. The decision is made in the processed signal to recover the binary sample bits which are then XORed with the bits in the same Lorenz sequence used during encryption to decrypt and retrieve the original “Plaintext Sequence”. Finally, an image can be obtained by the conversion and re-arrangement of the retrieved “Plaintext Sequence”.

**“*HELLO, THU!*” *Transmission:*** In this optical wireless communication demonstration, a string is carried by on-off keying (OOK) modulation signals transmitted by a 910 nm LED. With a pre-designed mapping schema where a single English alphabet or ASCII symbol maps to a unique 8-bit-sequence, the information sequence is generated and represents “HELLO, THU!” A pre-defined sequence for training the receiver's Least Mean Squares (LMS) equalizer is appended to it. Then before transmitted the signals, a synchronization sequence is added to the whole sequence for ease of synchronization and identification by the receiver.

Upon reception, synchronization, and identification, the receiver extracts both the information and training sequences. Initially, the equalizer is trained by the training sequence and applied to equalize the received information sequence. Subsequently, the equalized sequence experiences normalization, decision-making, and mapping to retrieve the original string.

***Statistical Analysis***

Statistical analysis was performed to ensure the reliability and significance of the reported data. The details are as follows:

Data Pre-processing: No pre-processing or transformation was applied to the raw data prior to statistical analysis. All data points were included in the analysis unless specifically noted as outliers, which were identified at a 90% confidence level.

Data Presentation: Data for device performance parameters (e.g., dark current density *J*_D_, responsivity *R*, detectivity *D**) obtained from multiple devices are presented as the mean ± standard deviation (SD). Representative data from a typical device are shown for *I*-*V* characteristics, I-t responses, and *EQE* spectra.

**Table S1** Fitting data of the TRPL decay for the perovskite film, perovskite film coated with PC_61_BM and BHJ.

| Sample | τ_1_ (ns) | τ_2_ (ns) | A_1_ (%) | A_2_ (%) | τ_avg_ (ns) |
| --- | --- | --- | --- | --- | --- |
| Perovskite | 4.25 | 106.52 | 14.03 | 85.97 | 105.85 |
| Perovskite /PC_61_BM | 1.28 | 10.15 | 87.46 | 15.54 | 6.46 |
| Perovskite /BHJ | 0.42 | 4.82 | 89.42 | 10.58 | 2.95 |

**Table S2** Fitted results of PPDs and PBPDs using revised diode equation (1).

|  | $\text{J}_{\text{0}}$ (A) | $\text{R}_{\text{S}}\text{ (Ω)}$ | $\text{n}$ | $\text{R}_{\text{s}\text{h}}$ (Ω) |
| --- | --- | --- | --- | --- |
| PPDs | 6.25×10^-9^ | 50.90 | 2.76 | 2.0×10^5^ |
| PBPDs | 7.14×10^-12^ | 1.01×10^3^ | 1.62 | 2.3×10^7^ |


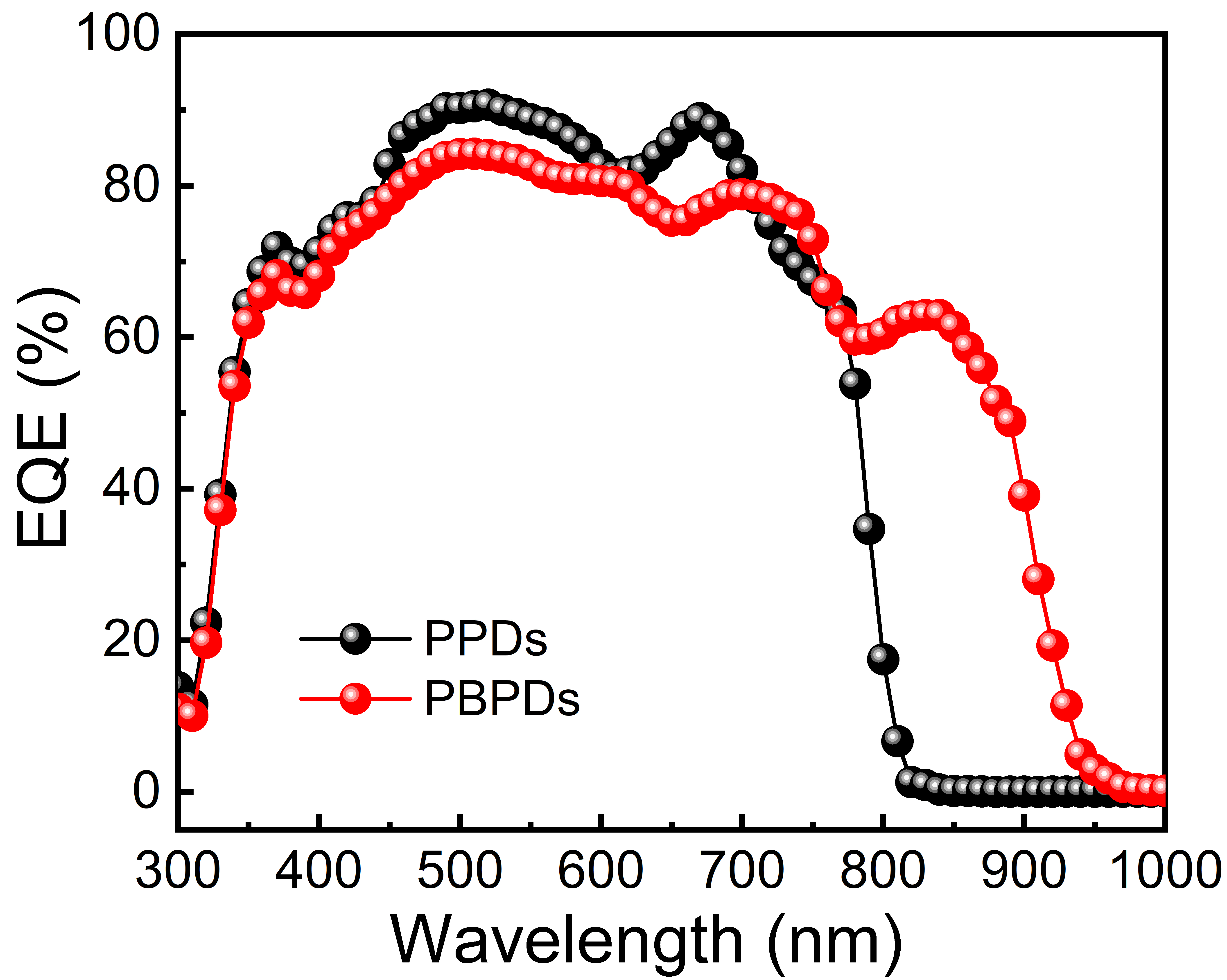


**Figure S1.** External quantum efficiency (*EQE*) of PPDs and PBPDs.


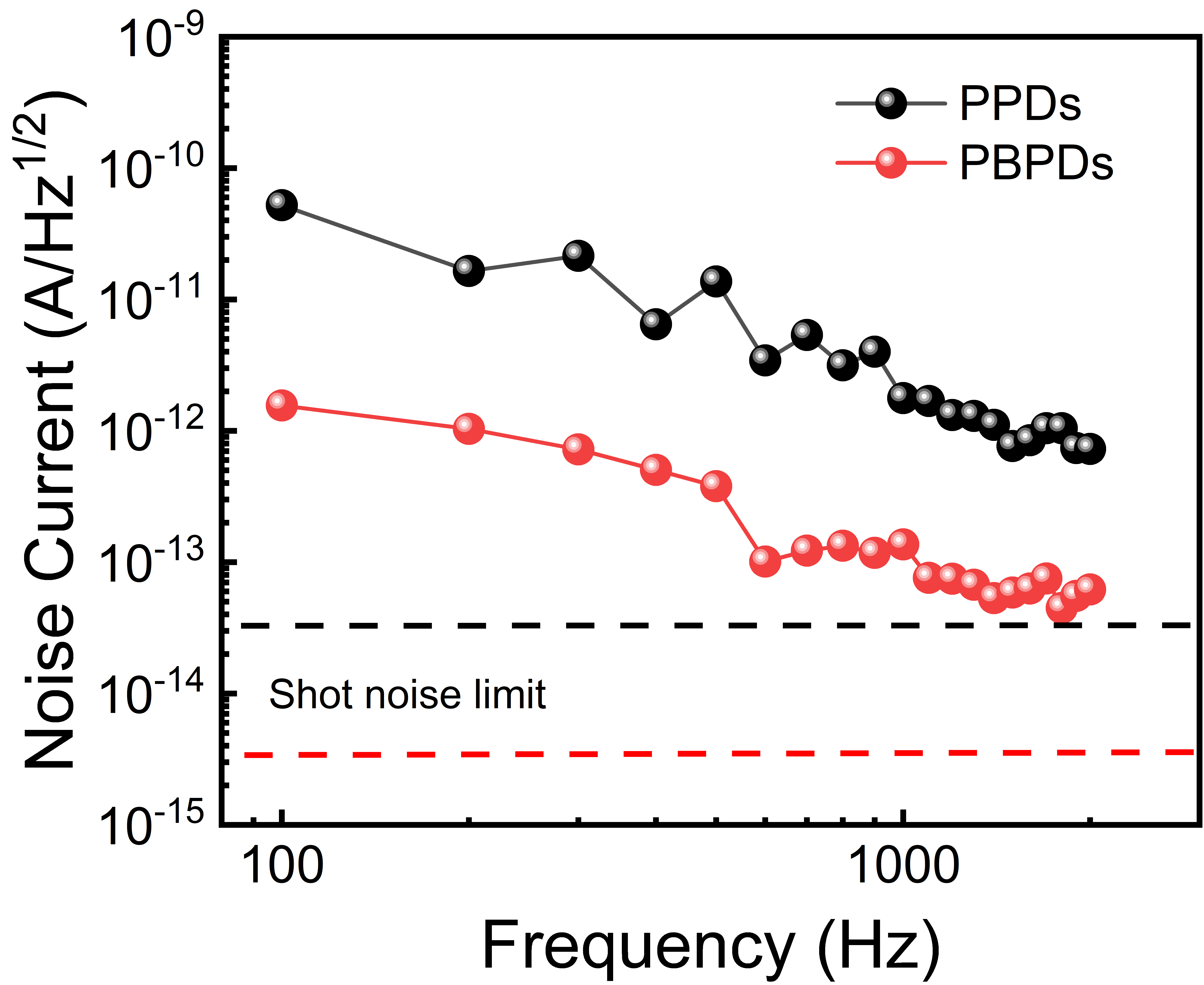


**Figure S2.** Noise spectral density of the PPDs and PBPDs under dark conditions at 0 V bias. The flat frequency response at higher frequencies indicates shot-noise-limited behavior. The significantly lower noise in PBPDs stems directly from its ultralow dark current (**Figure 2e**).

**
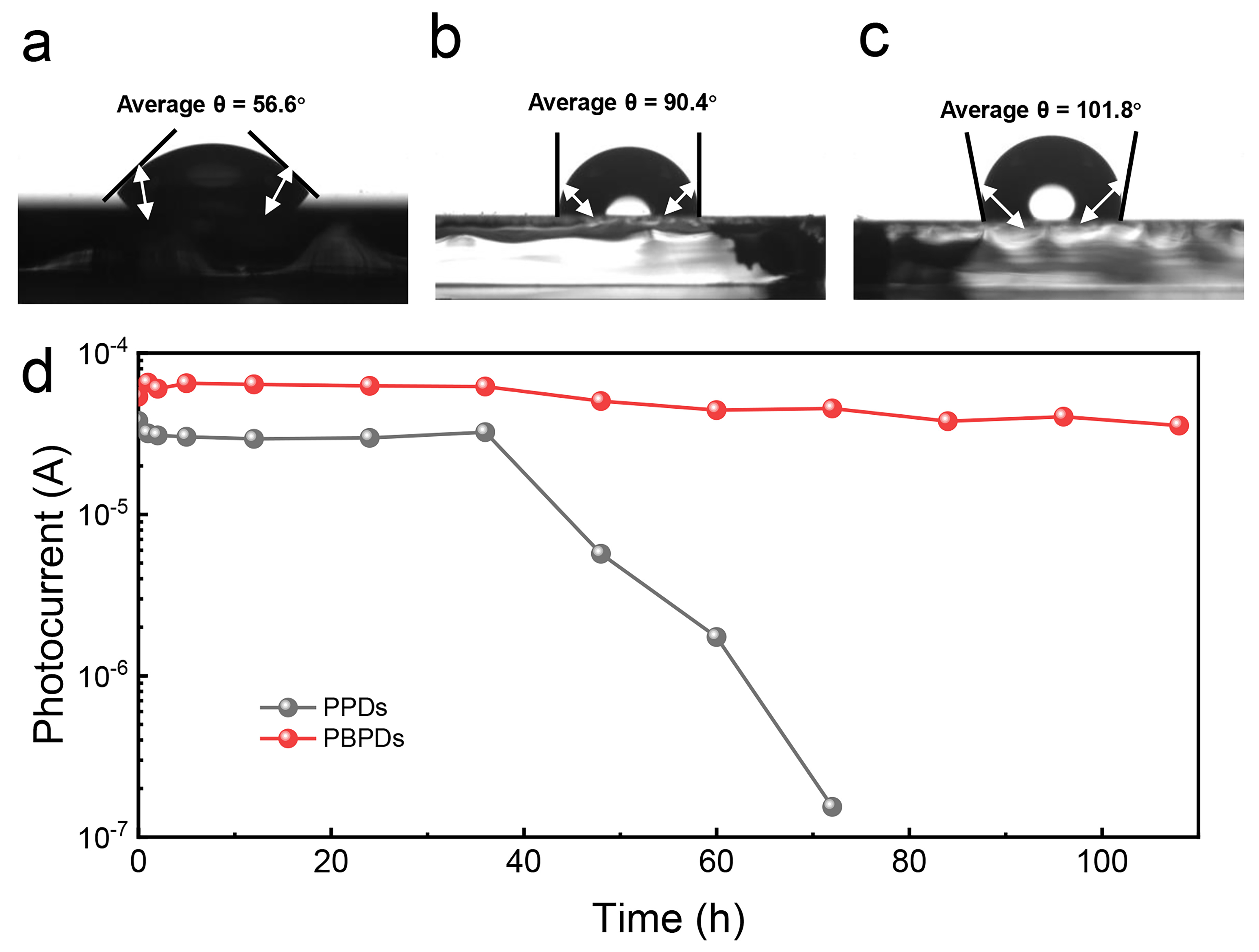
**

**Figure S3.** Contact angle measurement of water droplets on the films of pristine perovskite film (a), perovskite film with PC_61_BM on the top (b), and perovskite film with PC_61_BM: D18:BTP-4F on the top (c). (d) Photocurrent tracking of PPDs and PBPDs under air atmosphere (air humidity about 60%).

**
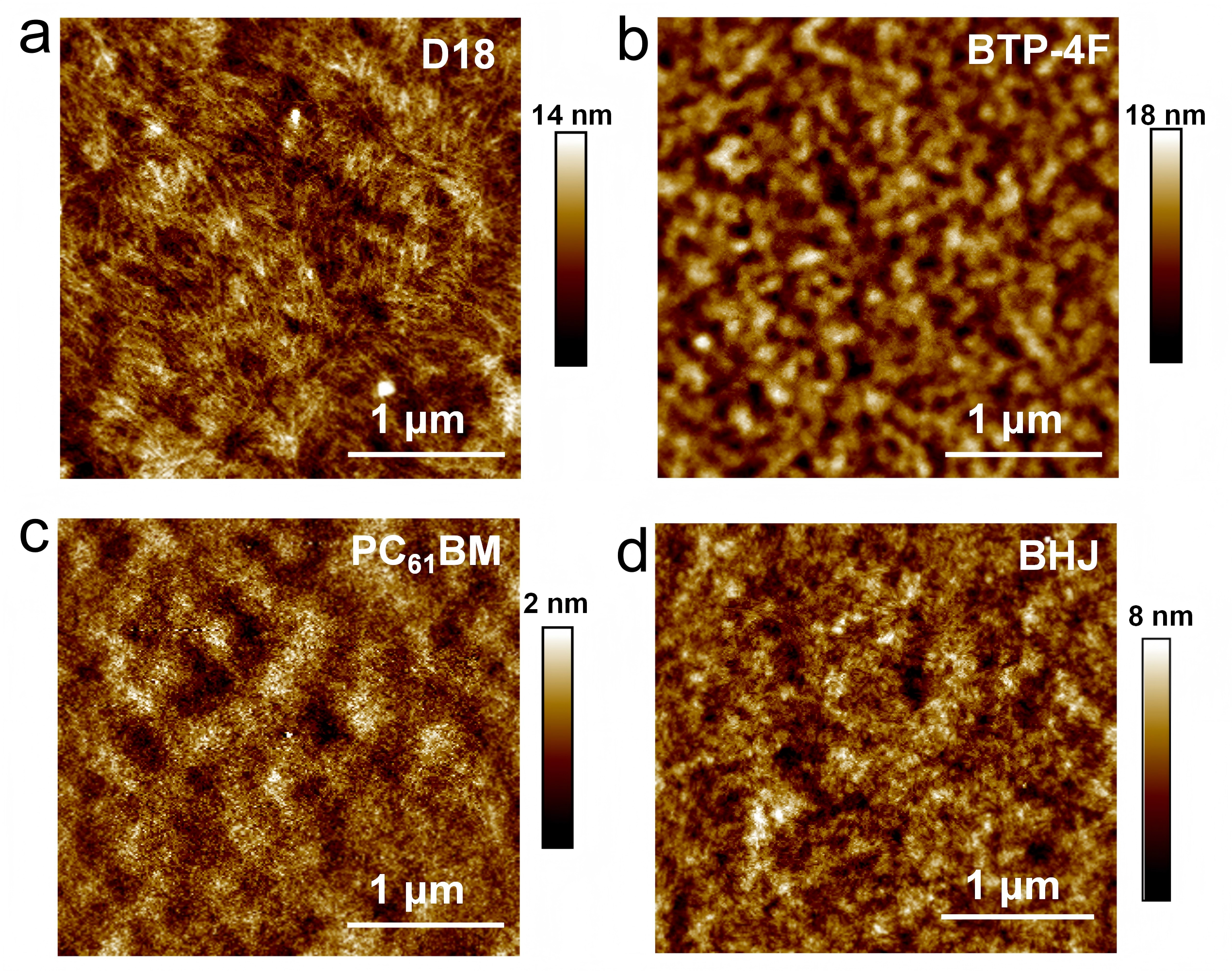
**

**Figure S4.** Atomic force microscopy (AFM) phase of D18 film, BTP-4F film, PC_61_BM film and BHJ film.


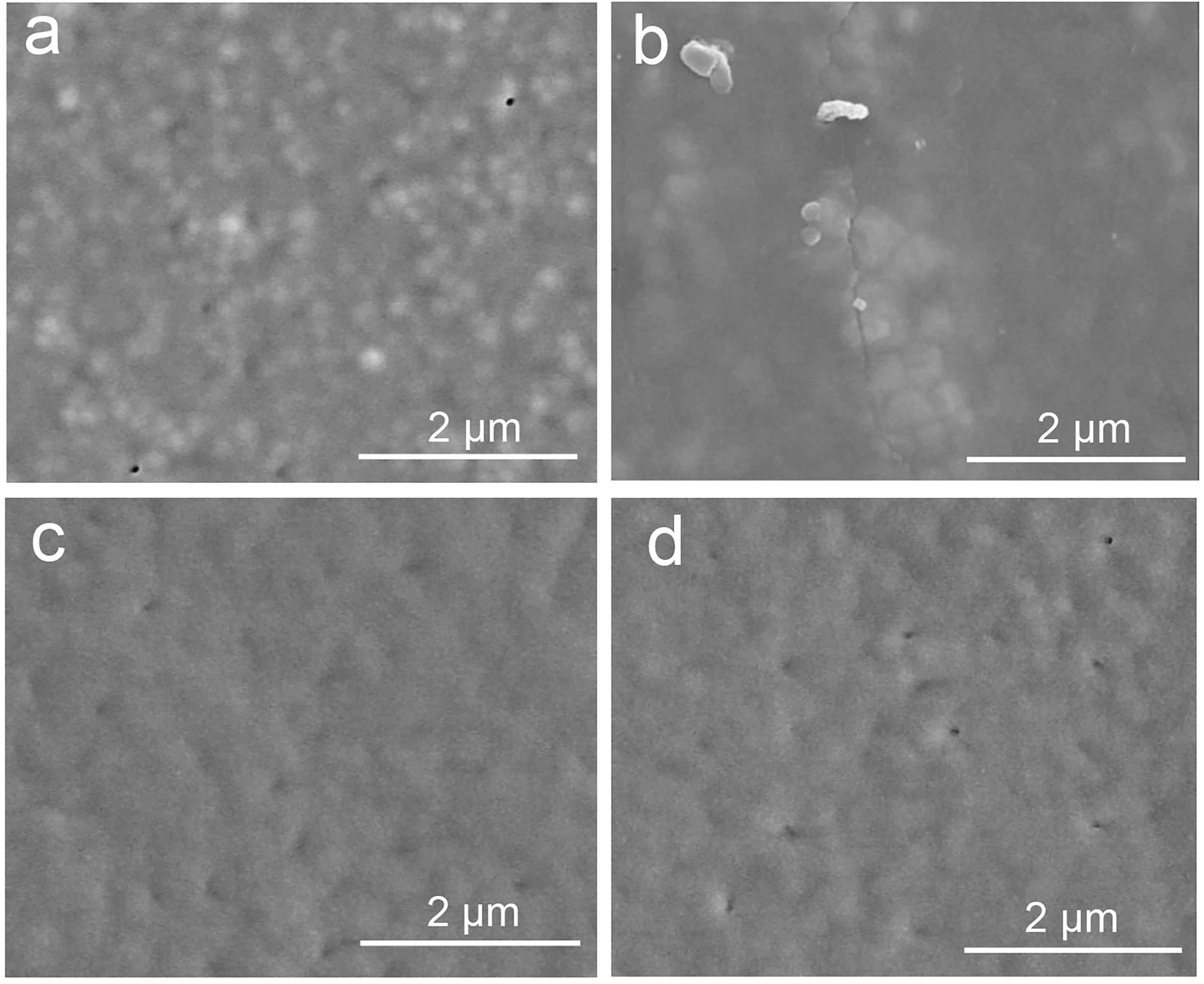


**Figure S5.** SEM images of perovskites with PC_61_BM(a), after the 1000 bending cycle tests for perovskite with PC_61_BM (b), perovskite with BHJ (c) and after the 1000 bending cycle tests for perovskite with BHJ (d) (the bending tests is for a curvature radius of 5 mm).

**
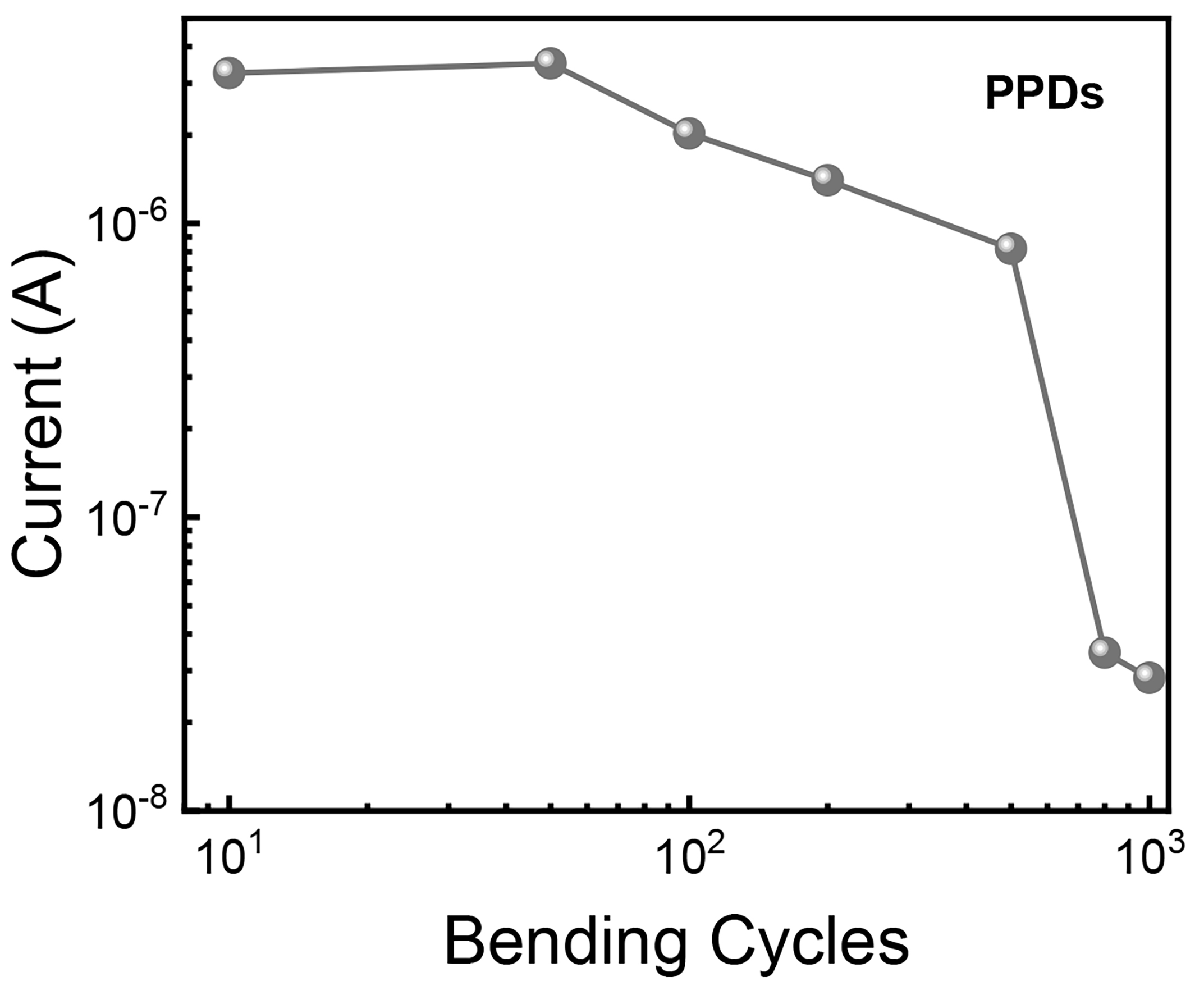
**

**Figure S6.** Photocurrent evolution of flexible PPDs as a function of mechanical bending cycles.


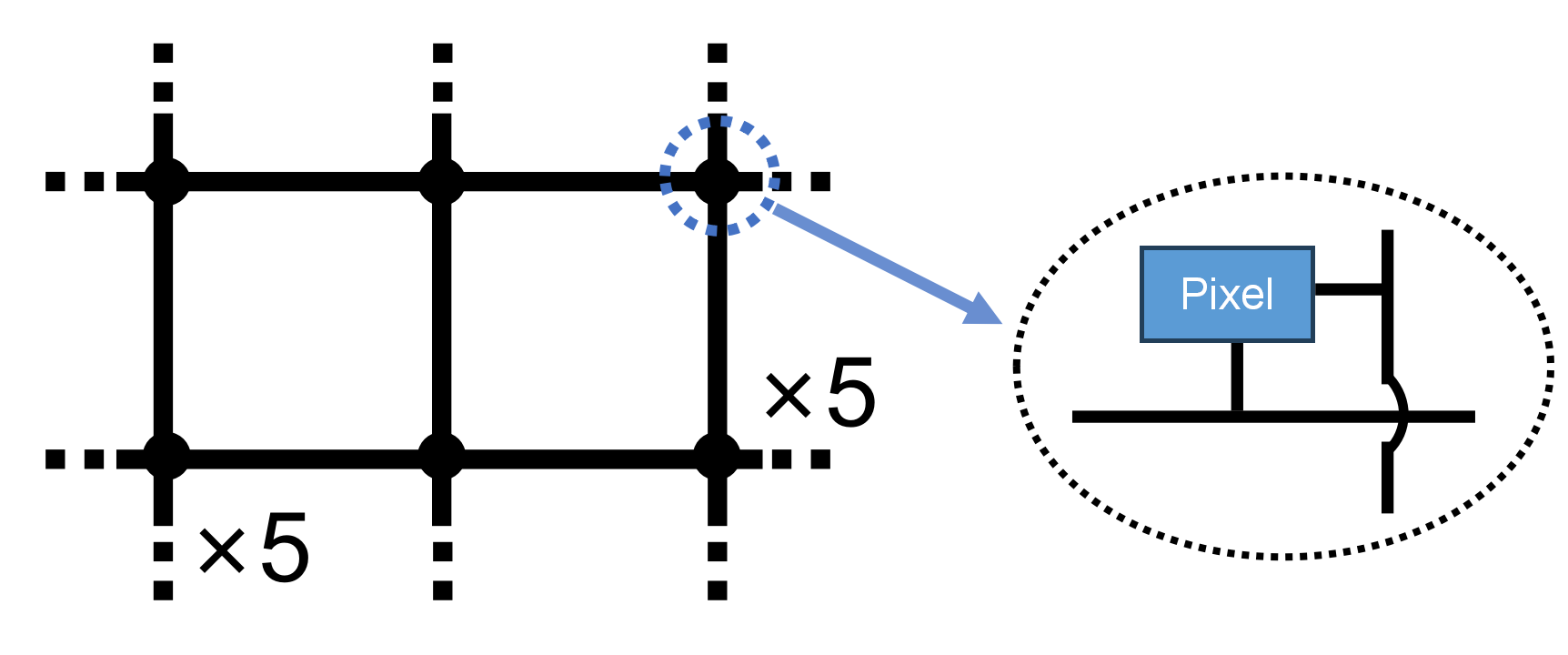


**Figure S7.** Schematic diagram of the 5×5 flexible photodetector array and the electrode addressing strategy.


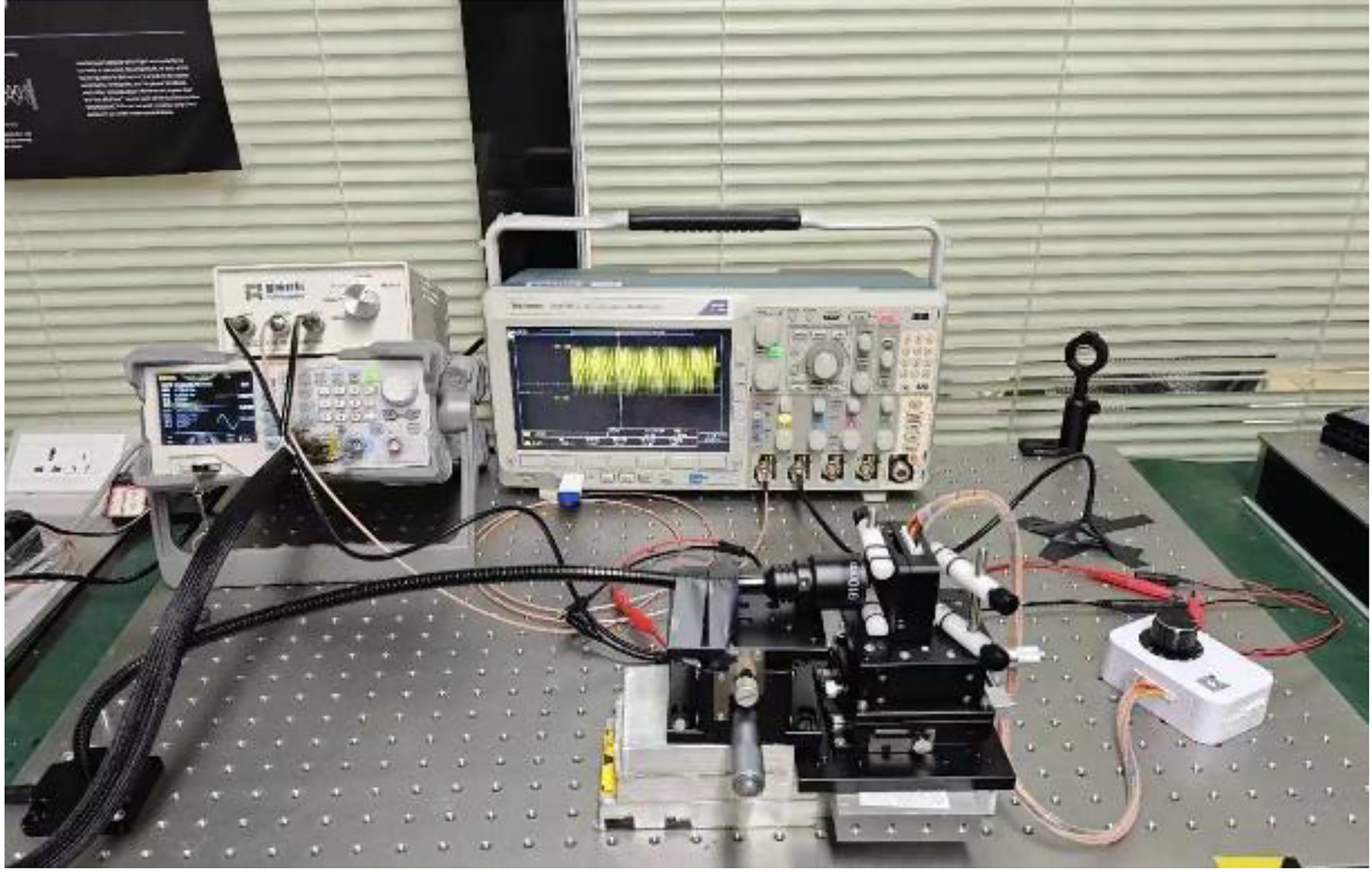


**Figure S8.** Physical diagram of picture information transmission process.
